# Supplementary material for: RUBCN as a novel prognostic biomarker and therapeutic target in breast cancer
Source: PLoS One. 2026 Jan 27;21(1):e0341357. doi: 10.1371/journal.pone.0341357 (PMC12843558; doi:10.1371/journal.pone.0341357)
Supplement: S1 Fig — (PDF) [file pone.0341357.s001.pdf]

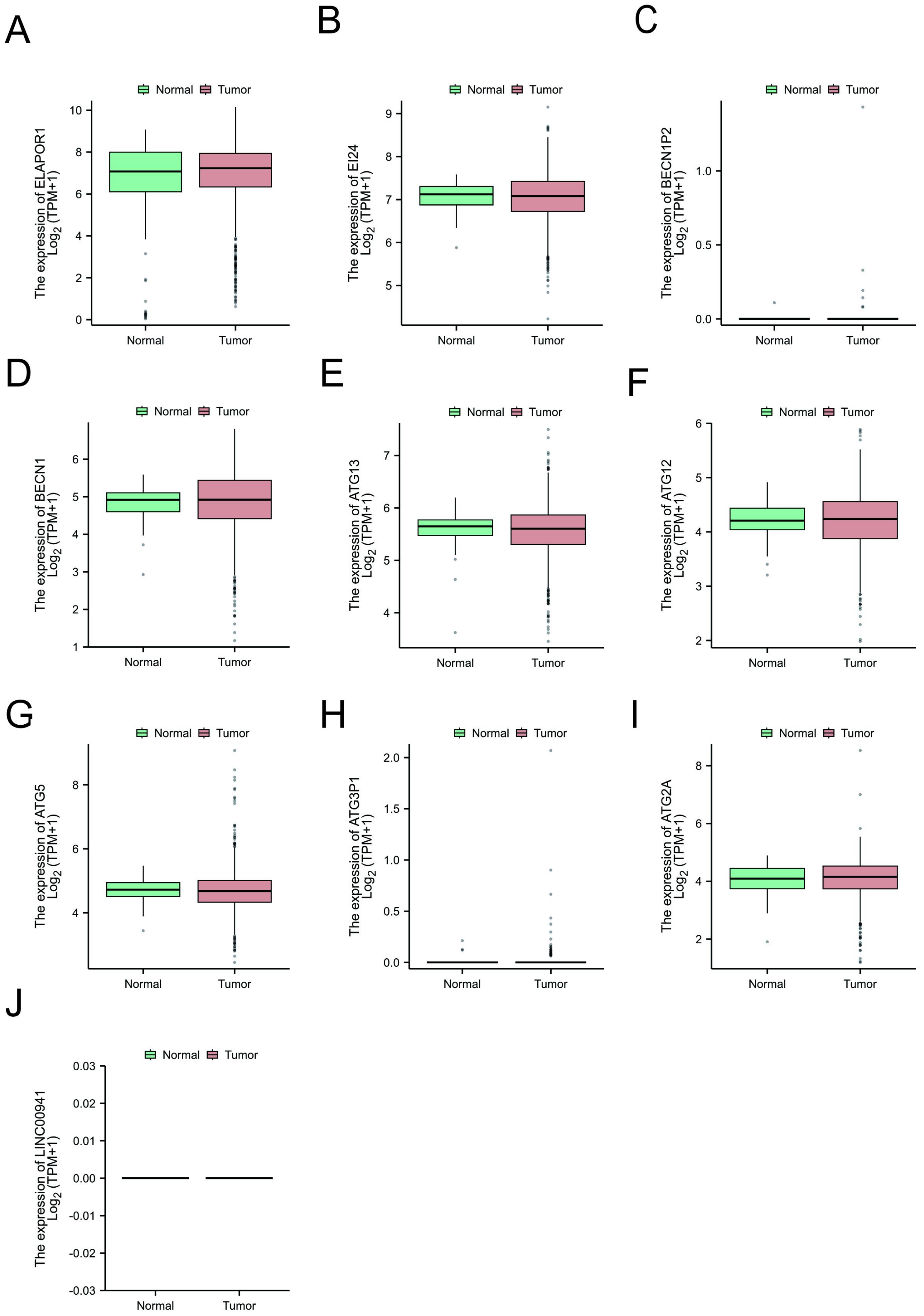


**S1 Fig**: Dysregulation of Core Autophagy Machinery in Breast Carcinogenesis. (A-J) Systematic comparison of mRNA expression for 10 key autophagy regulators between normal breast tissue and invasive ductal carcinoma.
