# Supplementary material for: RUBCN as a novel prognostic biomarker and therapeutic target in breast cancer
Source: PLoS One. 2026 Jan 27;21(1):e0341357. doi: 10.1371/journal.pone.0341357 (PMC12843558; doi:10.1371/journal.pone.0341357)
Supplement: S2 Fig — (A-K) Paired comparative analysis reveals dysregulation of 11 key autophagy-related genes (ARGs) in breast cancer. (PDF) [file pone.0341357.s002.pdf]

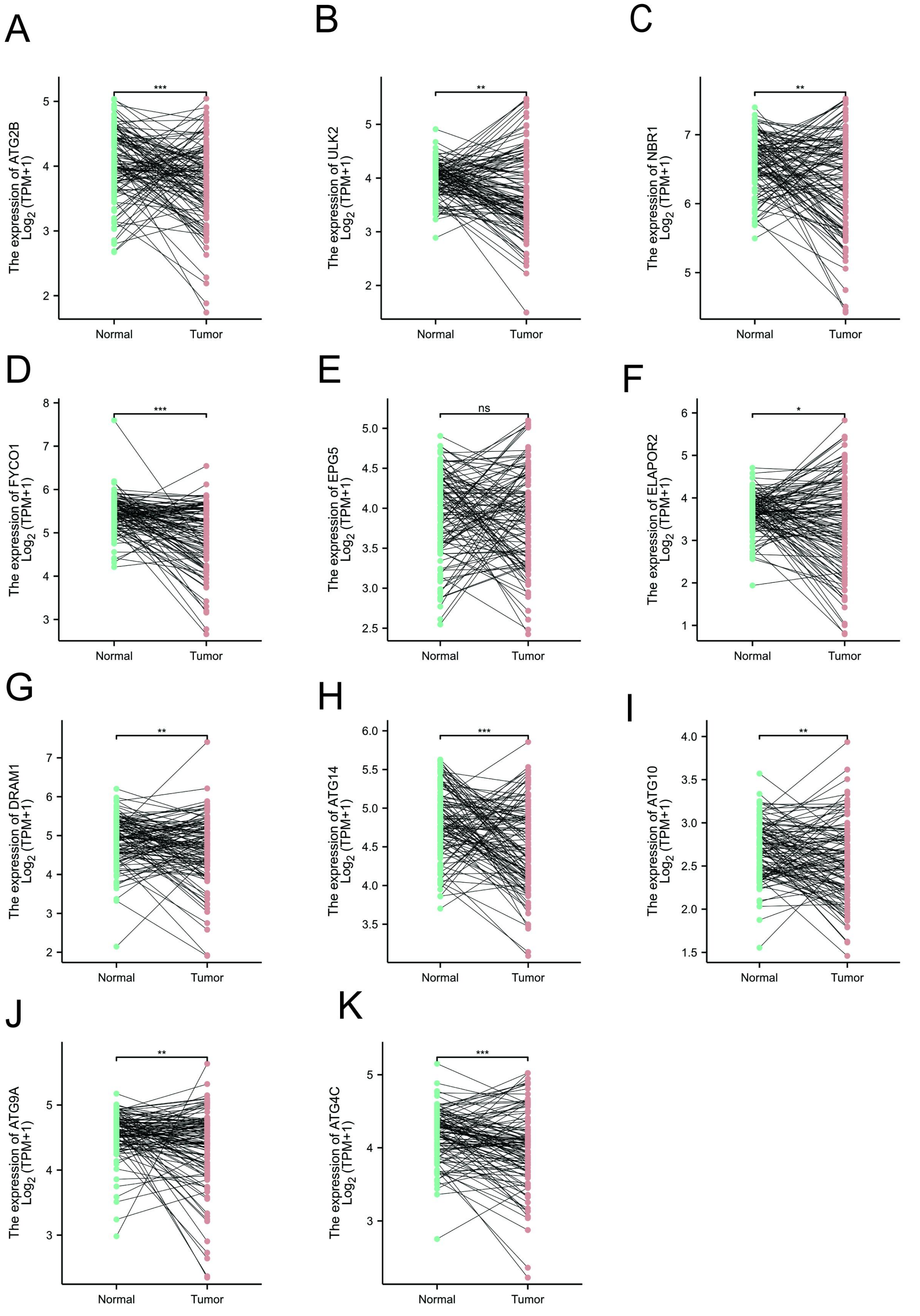

S2 Fig : Differential mRNA expression of core autophagy-related genes in matched normal and tumor breast tissues. (A-K) Paired comparative analysis reveals dysregulation of 11 key autophagy-related genes (ARGs) in breast cancer.
