# Supplementary material for: RUBCN as a novel prognostic biomarker and therapeutic target in breast cancer
Source: PLoS One. 2026 Jan 27;21(1):e0341357. doi: 10.1371/journal.pone.0341357 (PMC12843558; doi:10.1371/journal.pone.0341357)
Supplement: S3 Fig — (A-Q) Paired box-and-whisker plots comparing transcript levels of 18 core autophagy regulators between histologically normal breast tissues and matched breast tumor specimens from the TCGA-BRCA cohort. (PDF) [file pone.0341357.s003.pdf]

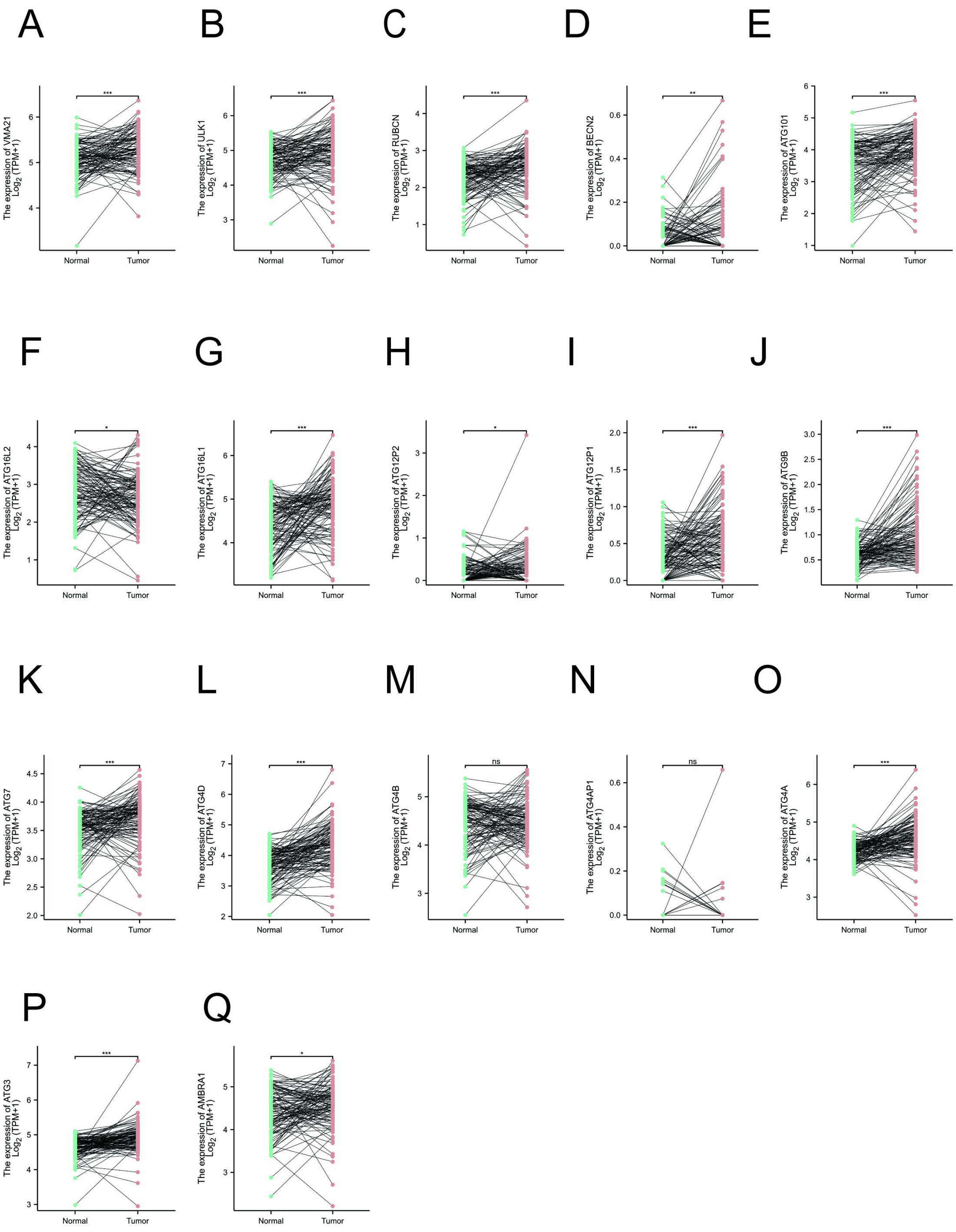

S3 Fig: Differential mRNA expression of 18 core autophagy-related genes in paired normal and neoplastic breast tissues. (A-Q) Paired box-and-whisker plots comparing transcript levels of 18 core autophagy regulators between histologically normal breast tissues and matched breast tumor specimens from the TCGA-BRCA cohort.
