# Supplementary material for: RUBCN as a novel prognostic biomarker and therapeutic target in breast cancer
Source: PLoS One. 2026 Jan 27;21(1):e0341357. doi: 10.1371/journal.pone.0341357 (PMC12843558; doi:10.1371/journal.pone.0341357)
Supplement: S1 Table — (PDF) [file pone.0341357.s004.pdf]

S1 Table. Primers used in quantitative real-time polymerase chain reaction

Gene	Forward primer	Reverse primer
**Rubcn**	5′-GATTACTGGCAGTTCGTGAAAGA-3′	5’-CTGCTCTGGTCGTTCTCGTG-3′
**β-Actin**	5′-TTAAGGAGAAGCTGTGCTACG-3′	5′-GTTGAAGGTAGTTTCGTGGAT-3′
