# Supplementary material for: RUBCN as a novel prognostic biomarker and therapeutic target in breast cancer
Source: PLoS One. 2026 Jan 27;21(1):e0341357. doi: 10.1371/journal.pone.0341357 (PMC12843558; doi:10.1371/journal.pone.0341357)
Supplement: S2 Table — (PDF) [file pone.0341357.s005.pdf]

S2 Table. The primer sequences information of Rubcn siRNA.

Name	primer sequences	
**siRUBCN#1**	GCAGUCUCAAACGGCAAAUTT	AUUUGCCGUUUGAGACUGCTT
**siRUBCN#2**	GCACAAAGAAGAGCCAUAUTT	AUAUGGCUCUUCUUUGUCCTT
**siCtrl**	UUCUCCGAACGUGUCACGUTT	ACGUGACACGUUCGGAGAATT
