# Supplementary material for: RUBCN as a novel prognostic biomarker and therapeutic target in breast cancer
Source: PLoS One. 2026 Jan 27;21(1):e0341357. doi: 10.1371/journal.pone.0341357 (PMC12843558; doi:10.1371/journal.pone.0341357)
Supplement: S3 Table — (PDF) [file pone.0341357.s006.pdf]

S3 Table: Autophagy-related genes were curated from the HUGO Gene Nomenclature Committee (HGNC) database (https://www.genenames.org/).

ATG2A
ATG2B
ATG3
ATG5
ATG7
ATG9A
ATG9B
ATG10
ATG12
ATG13
ATG14
ATG101
RUBCN
CERKL
DEPP1
NBR1
RUBCNL
AMBRA1
ATG3P1
ATG4A
ATG4B
ATG4C
ATG4D
ATG12P1
ATG12P2
ATG16L1
ATG16L2
EI24
DRAM1
DRAM2
ATG4AP1
ELAPOR1
EPG5
FYCO1
ULK1
ULK2
ELAPOR2
SQSTM1
BECN1P2

BECN2
