# Supplementary material for: RUBCN as a novel prognostic biomarker and therapeutic target in breast cancer
Source: PLoS One. 2026 Jan 27;21(1):e0341357. doi: 10.1371/journal.pone.0341357 (PMC12843558; doi:10.1371/journal.pone.0341357)

Use Thermo Scientific™ PageRuler™ Prestained Protein Ladder, catalog number 26616, a protein molecular weight standard ranging from 10 to 180 kDa. The molecular weights from top to bottom are 180 kDa, 130 kDa, 100 kDa, 70 kDa, 55 kDa, 40 kDa, 35 kDa, 25 kDa, 15 kDa, and 10 kDa. After exposure, elute using Kangwei Century Stripping Buffer. The item number is CW0056M

Figure_2B_RUBCN(MCF-10A,MCF-7,MDA-MB-468,MDA-MB-231,SK-BR-3)

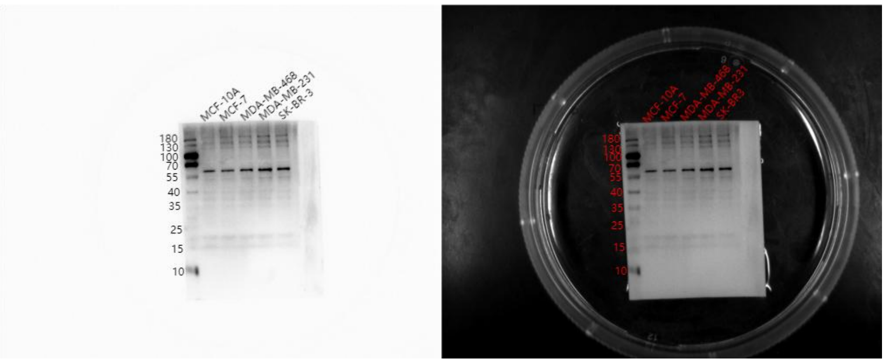

Figure_2B_GAPDH(MCF-10A,MCF-7,MDA-MB-468,MDA-MB-231,SK-BR-3)

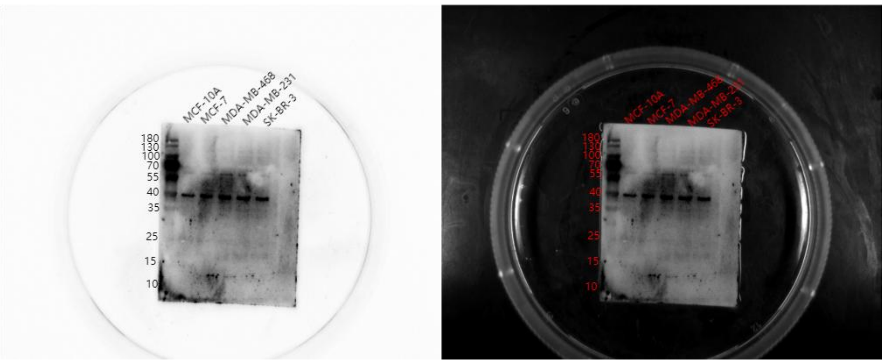

Figure_4D_MDA-MB-231_GAPDH(left,Vector,middle,siRUBCN#1,right,siRUBCN

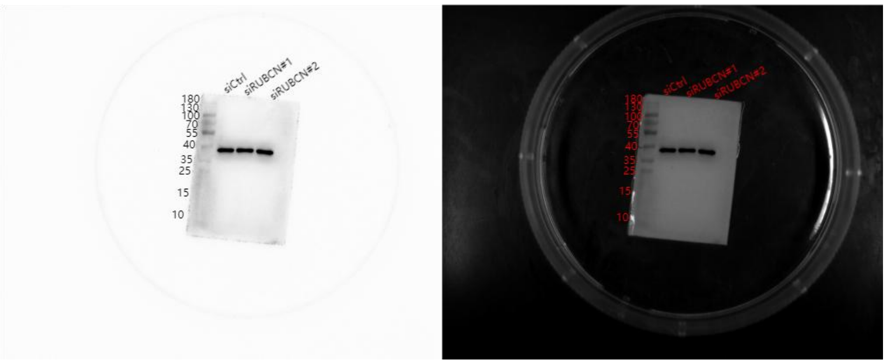

#2)

Figure_4D_MDA-MB-231_RUBCN(left,Vector,middle,siRUBCN#1,right,siRUBCN

#2)

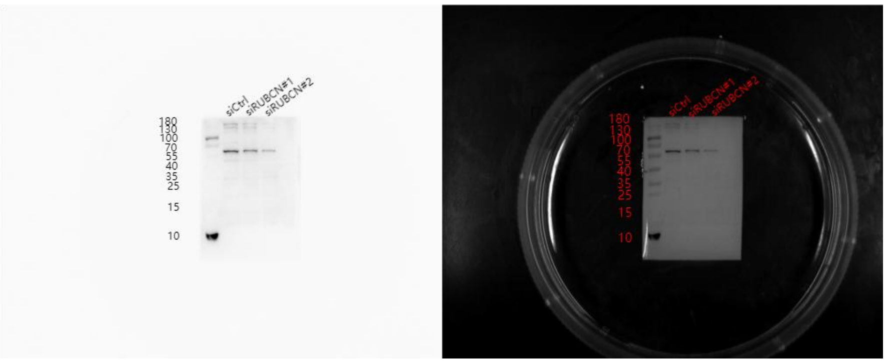

Figure_9E_MDA-MB-231_LC3 (left, Vector, right, siRUBCN, left, Vector, right,siRUBCN+CQ)

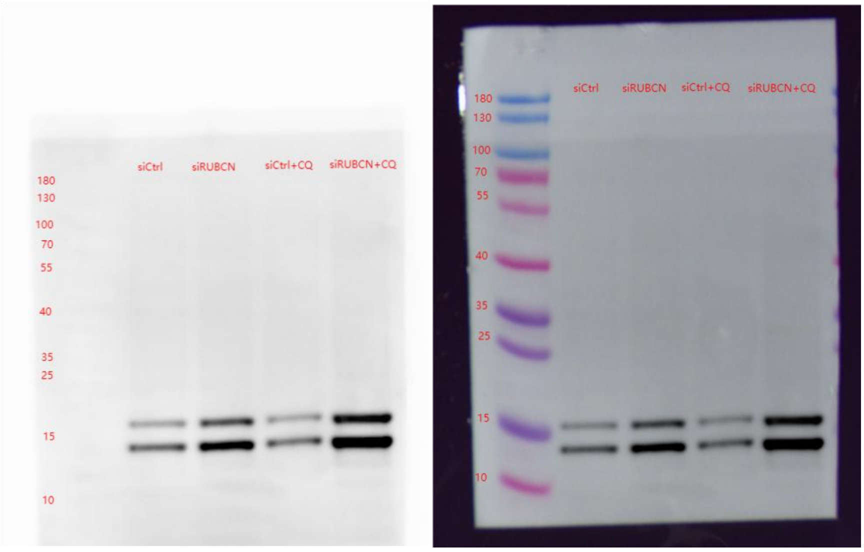

Figure_9E_MDA-MB-231_P62 (left, Vector, right, siRUBCN, left, Vector, right,siRUBCN+CQ)

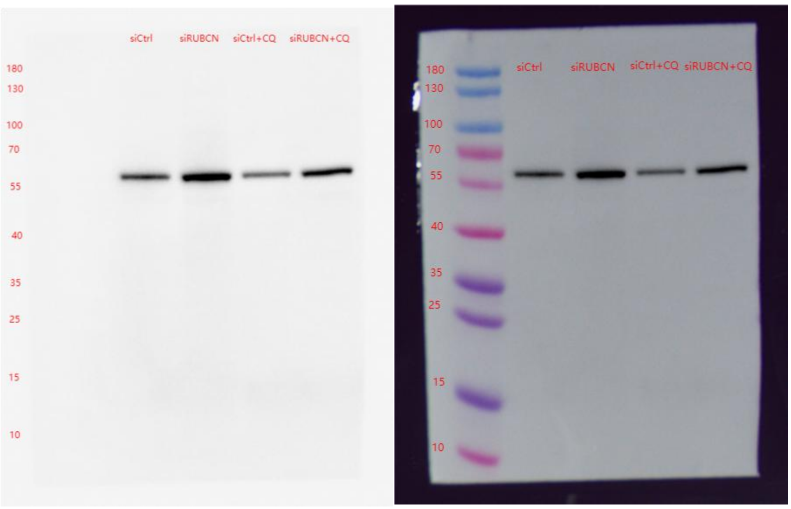

Figure_9E_MDA-MB-231_GAPDH (left, Vector, right, siRUBCN, left, Vector, right,siRUBCN+CQ)

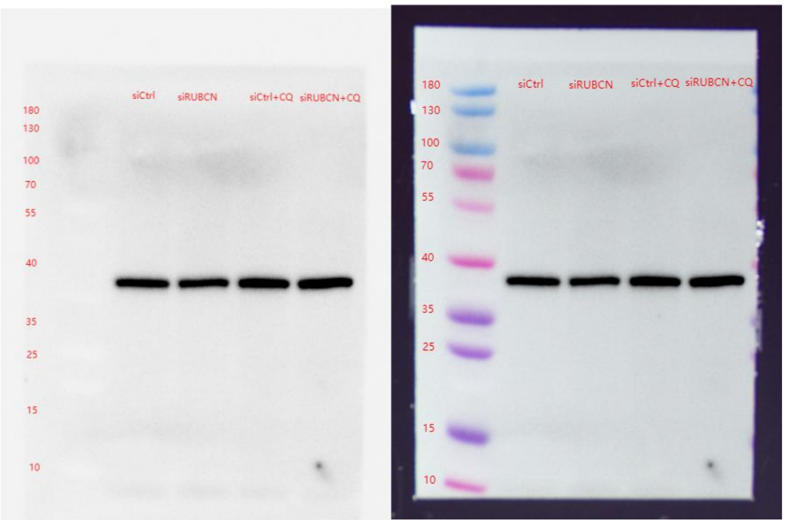

Figure_9E_MDA-MB-231_RUBCN (left, Vector, right, siRUBCN, left, Vector, right,siRUBCN+CQ)

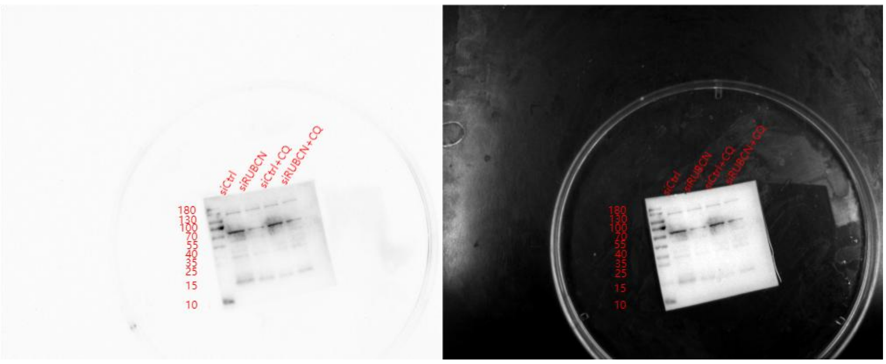

Figure_9E_MDA-MB-231_GAPDH (left, Vector, right, siRUBCN, left, Vector, right,siRUBCN+CQ)

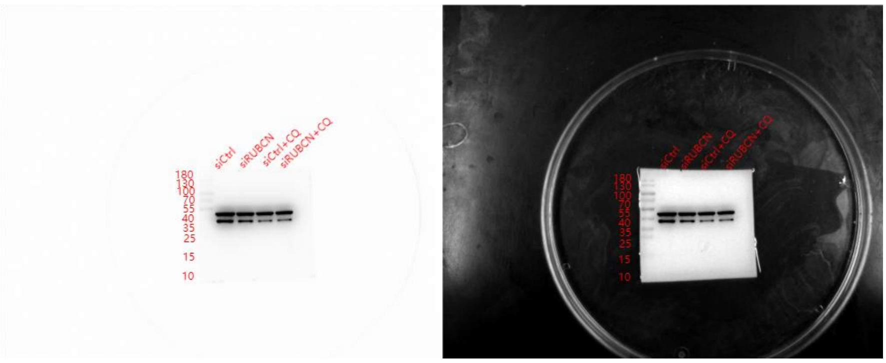

Supplement: S1 Data — (PDF) [file pone.0341357.s007.pdf]
