# Supplementary figures and images for: RUBCN as a novel prognostic biomarker and therapeutic target in breast cancer
Source: PLoS One. 2026 Jan 27;21(1):e0341357. doi: 10.1371/journal.pone.0341357 (PMC12843558; doi:10.1371/journal.pone.0341357)

Figure_3C_Normal(100x,400x)


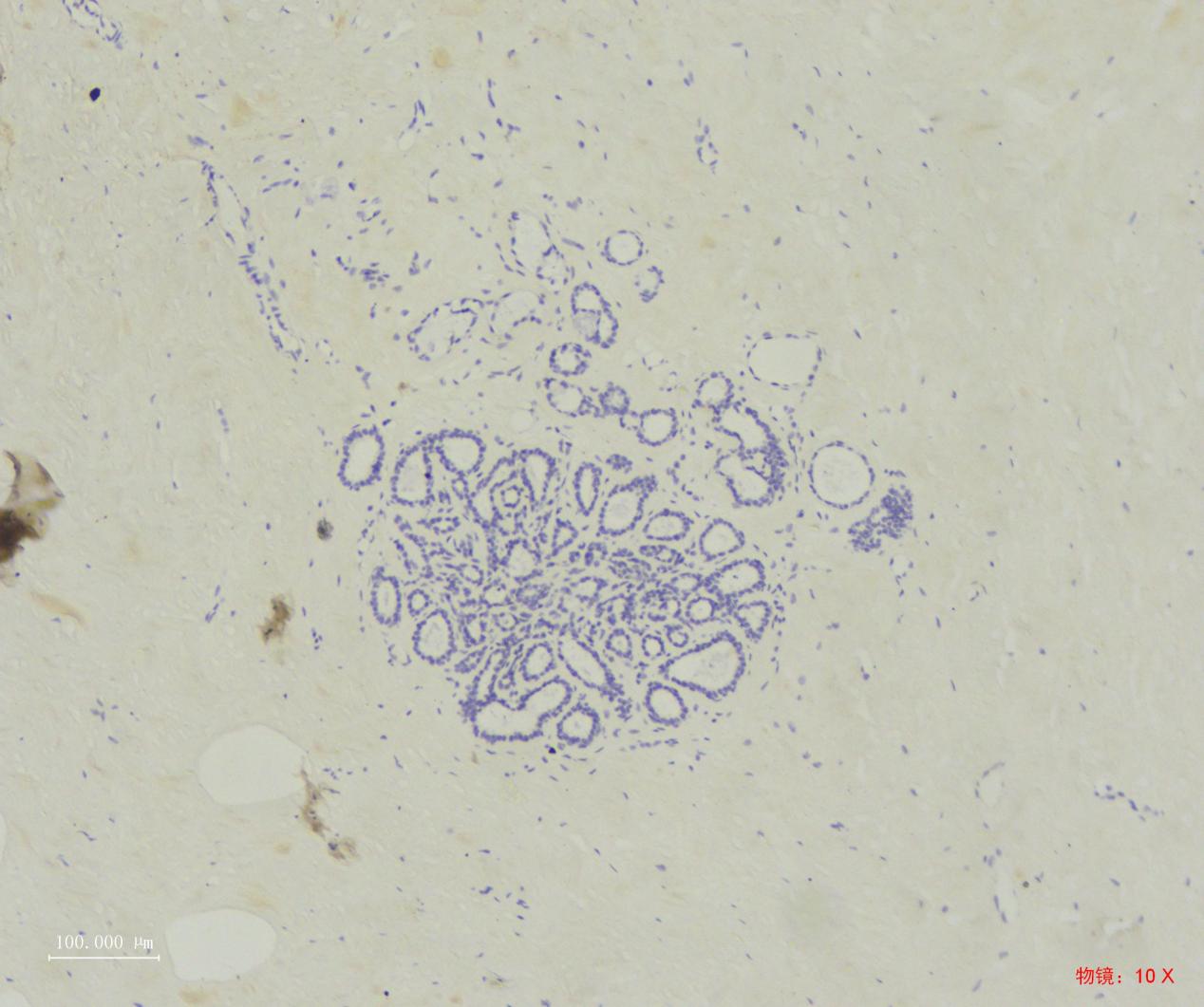

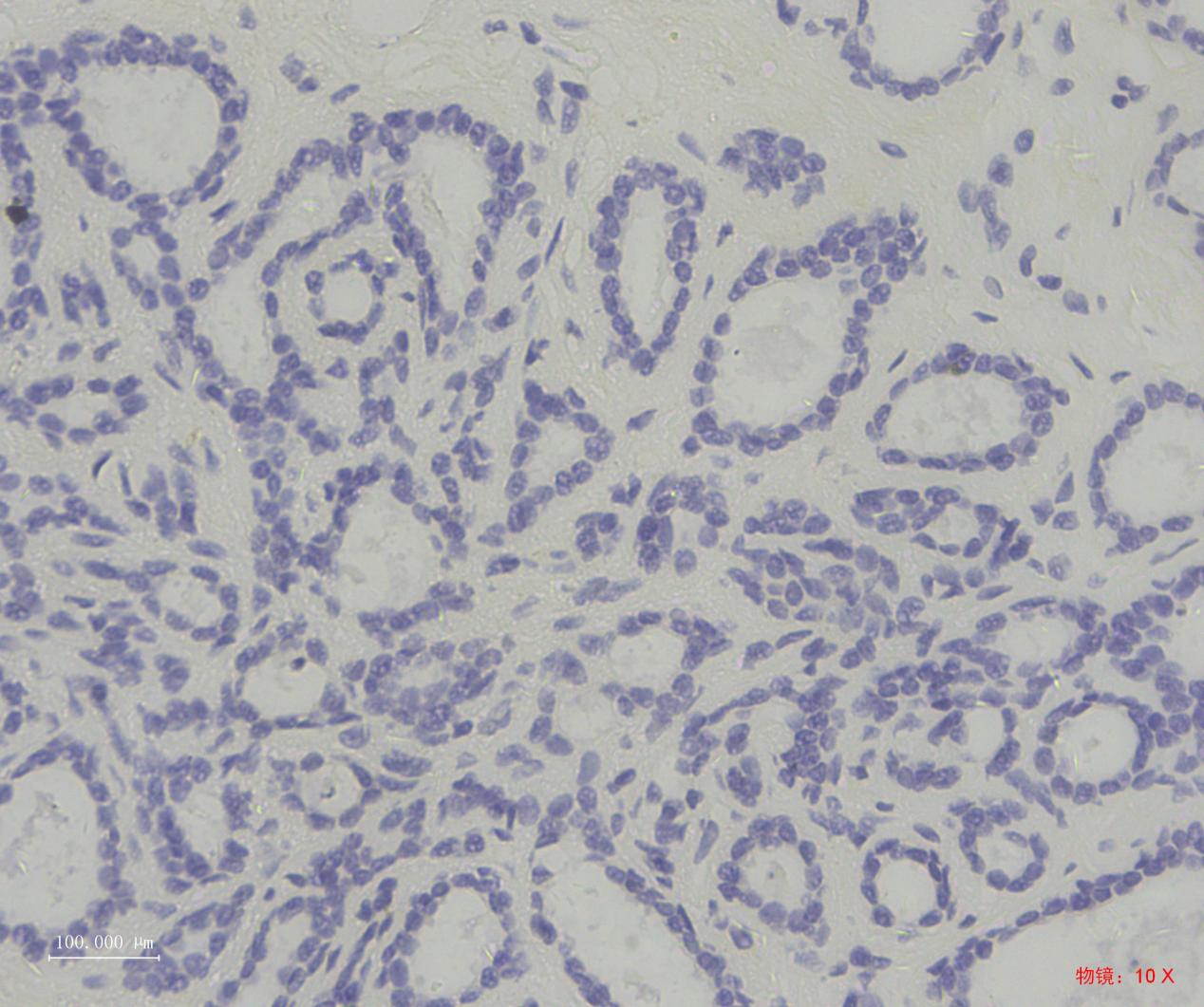

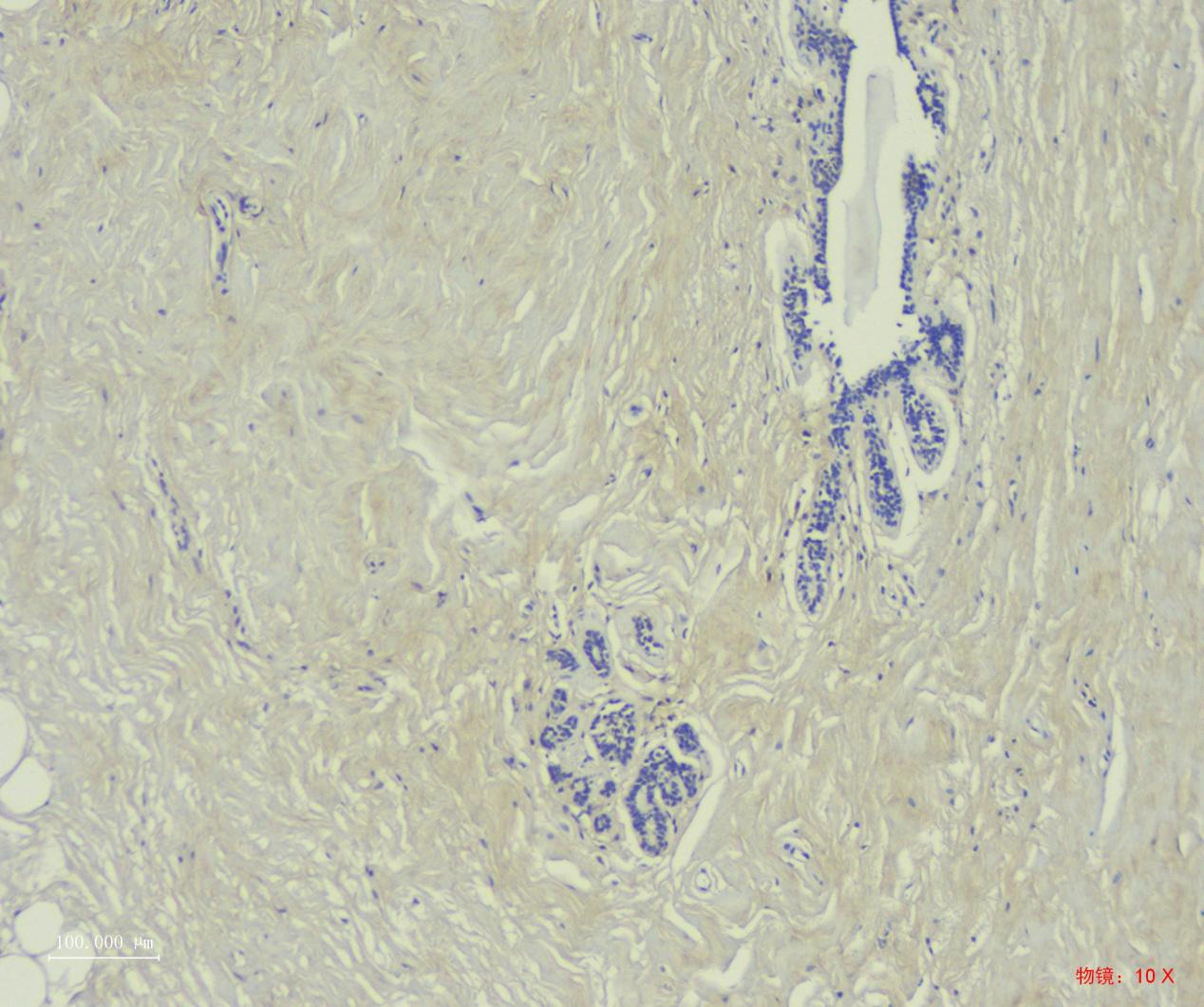

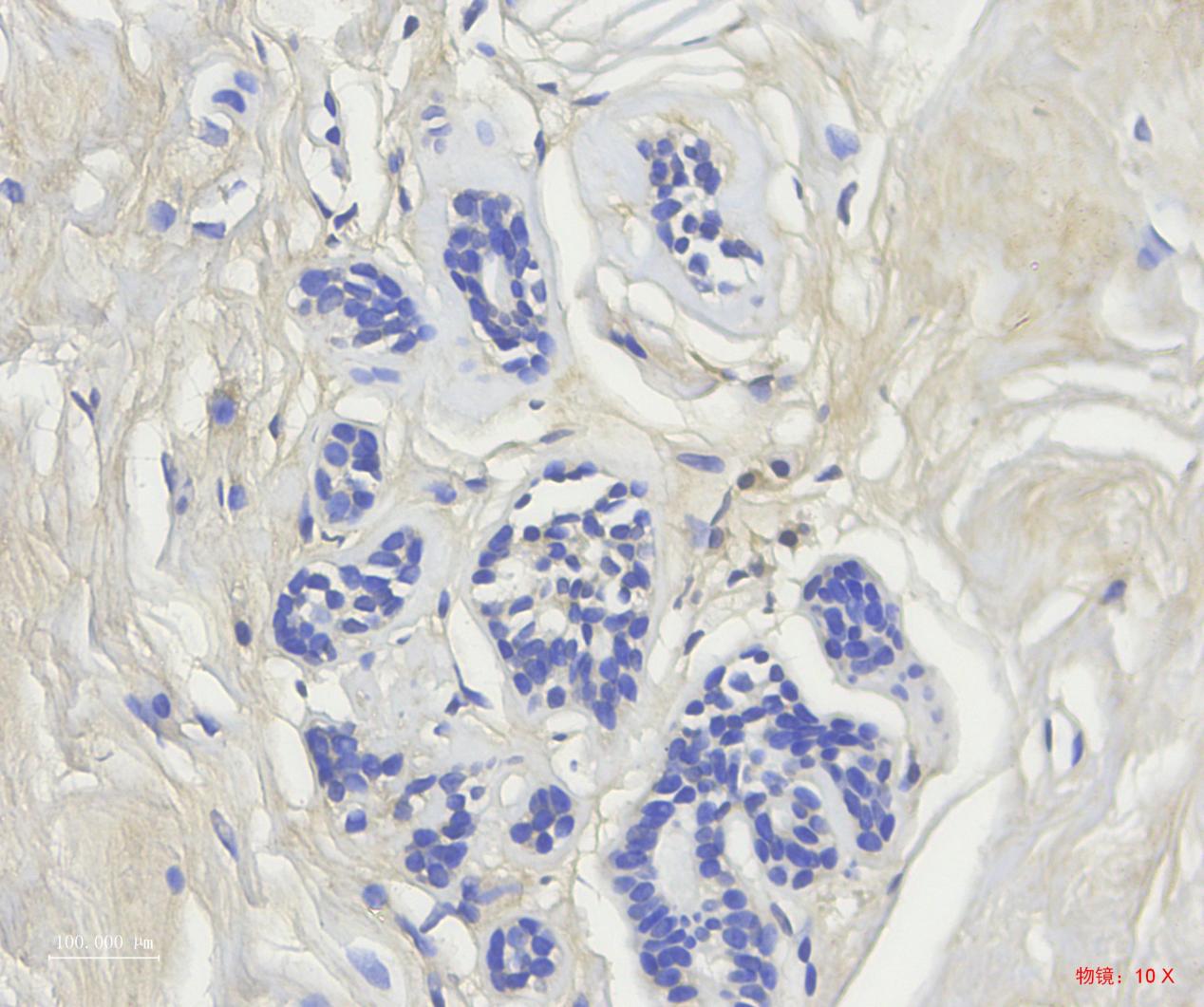

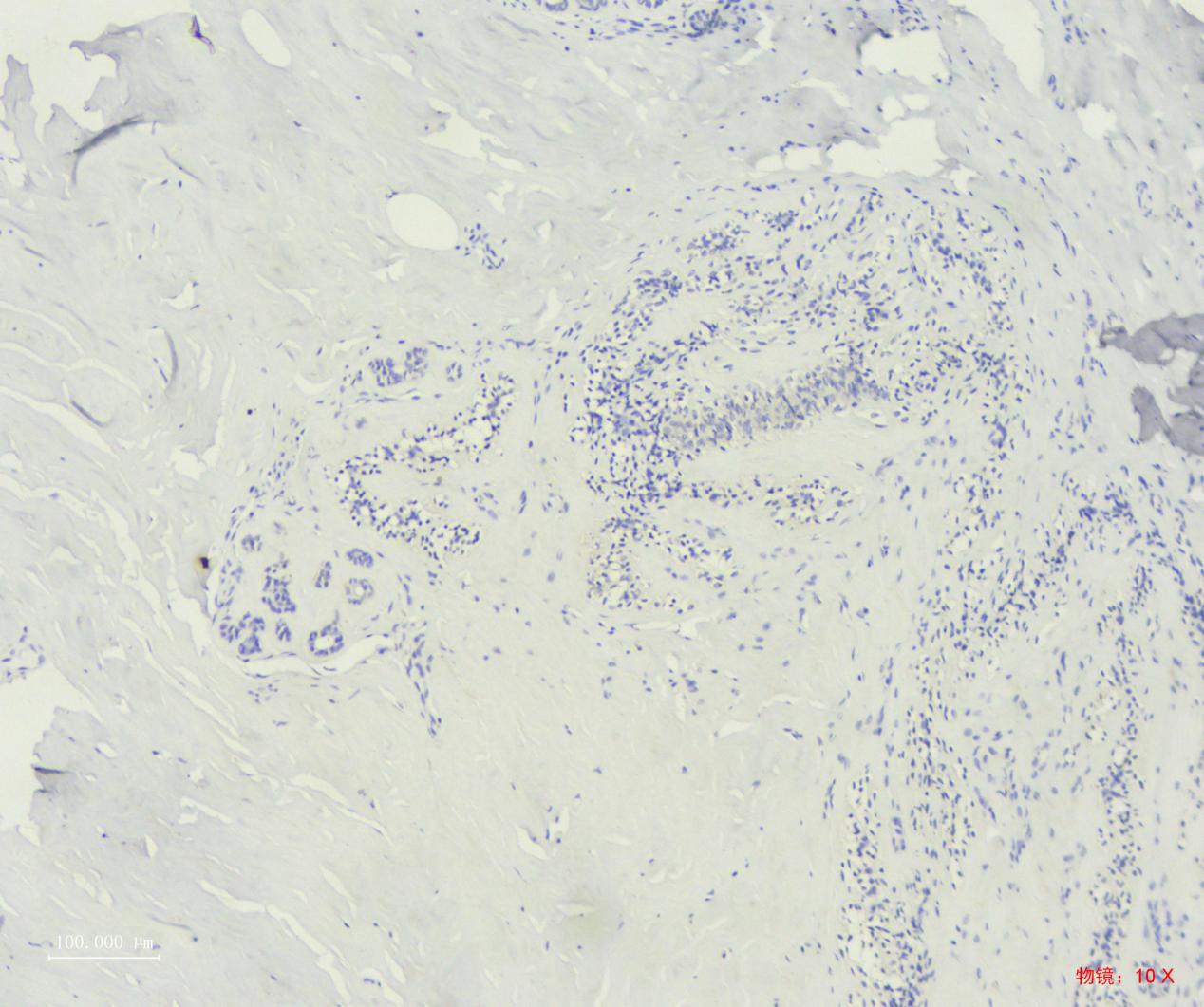

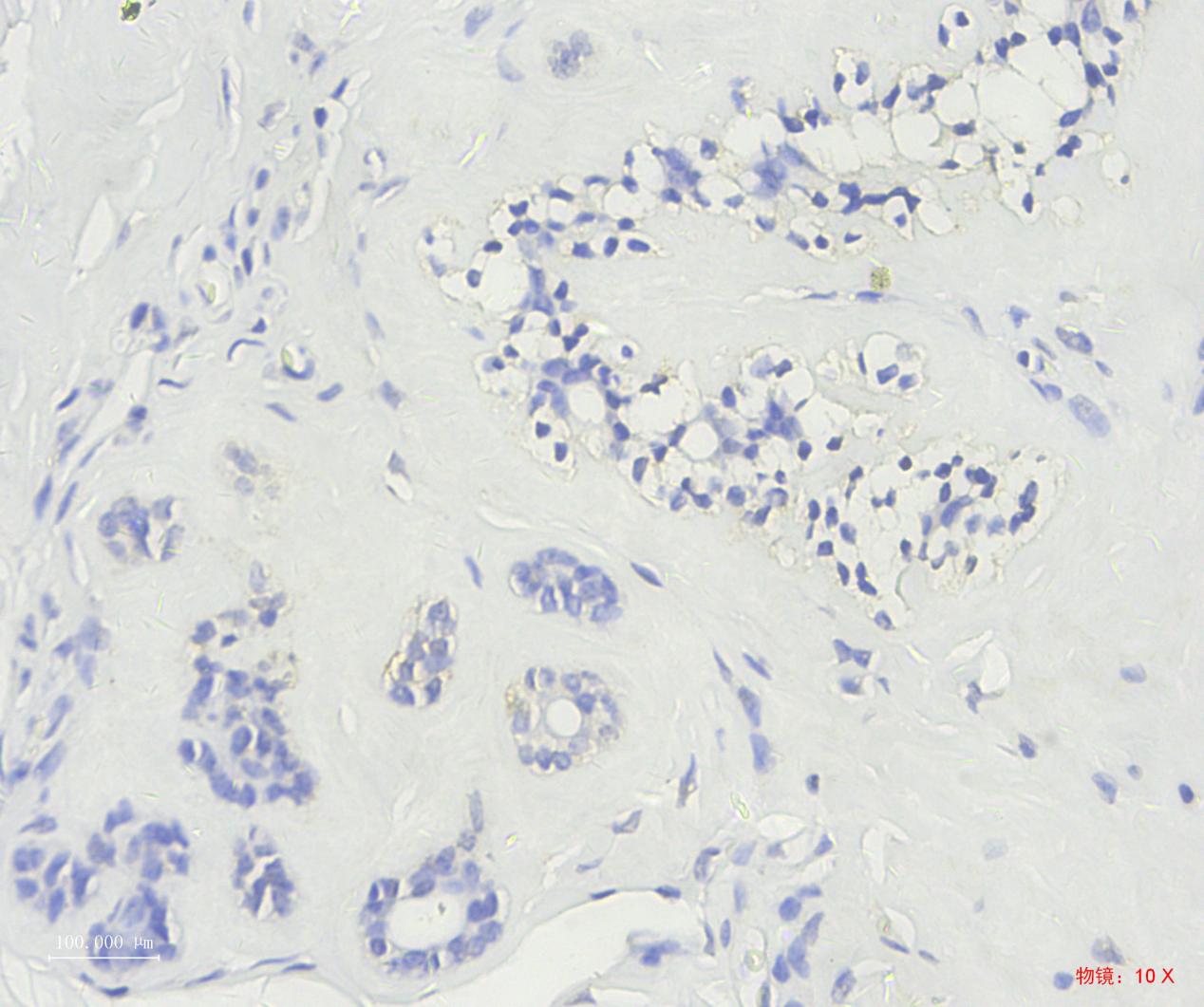


Figure_3C_Tumor(100x,400x)


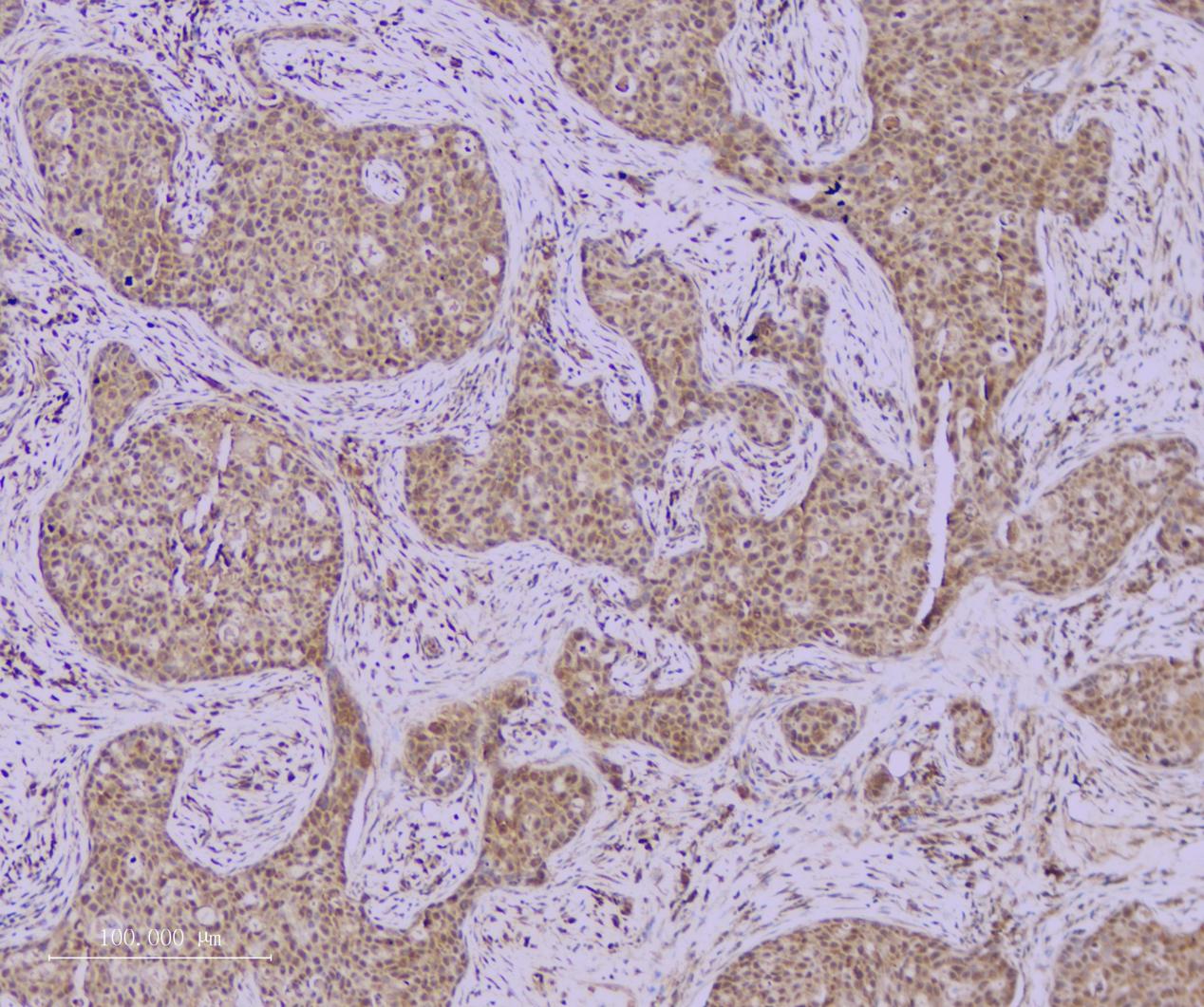

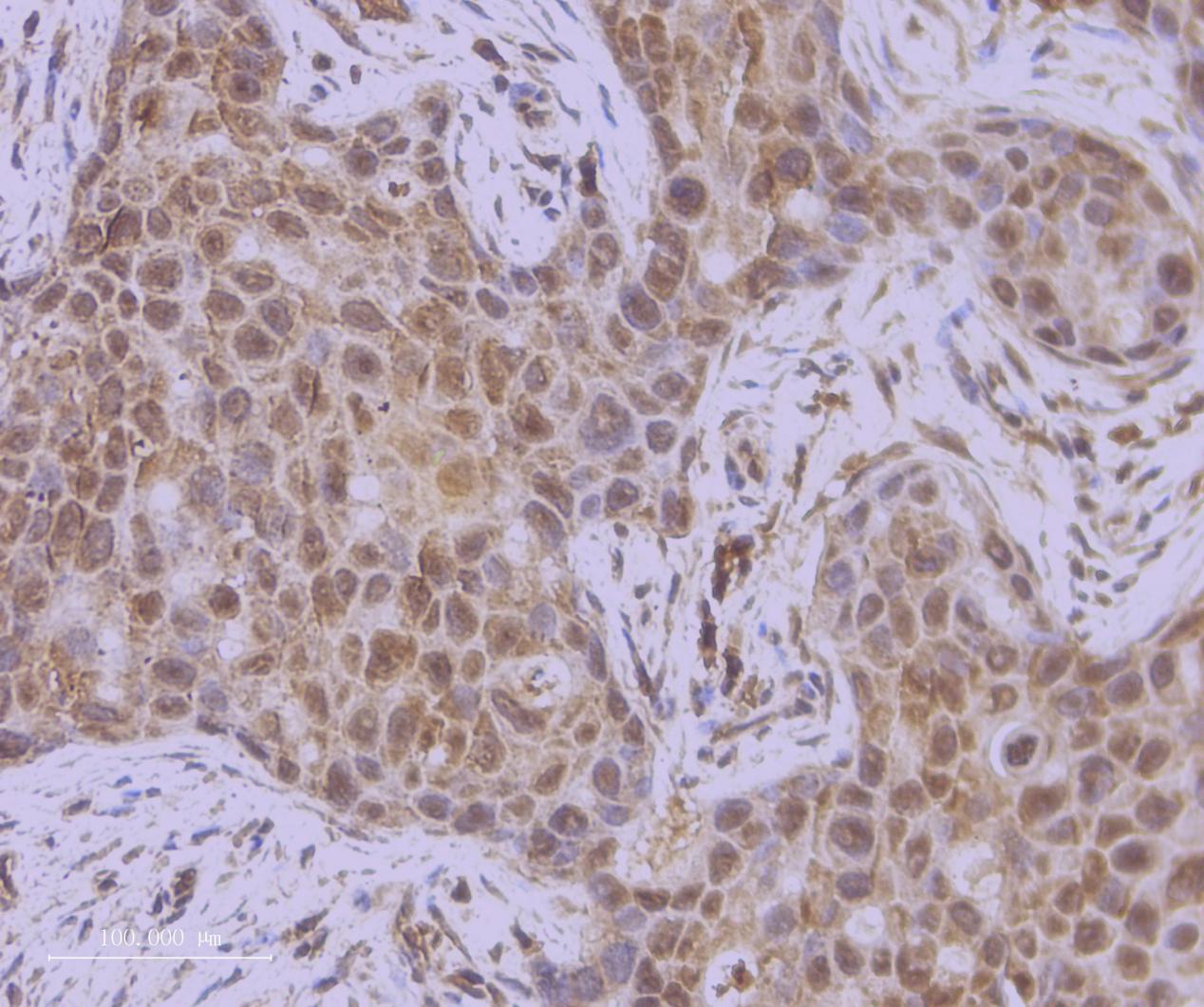

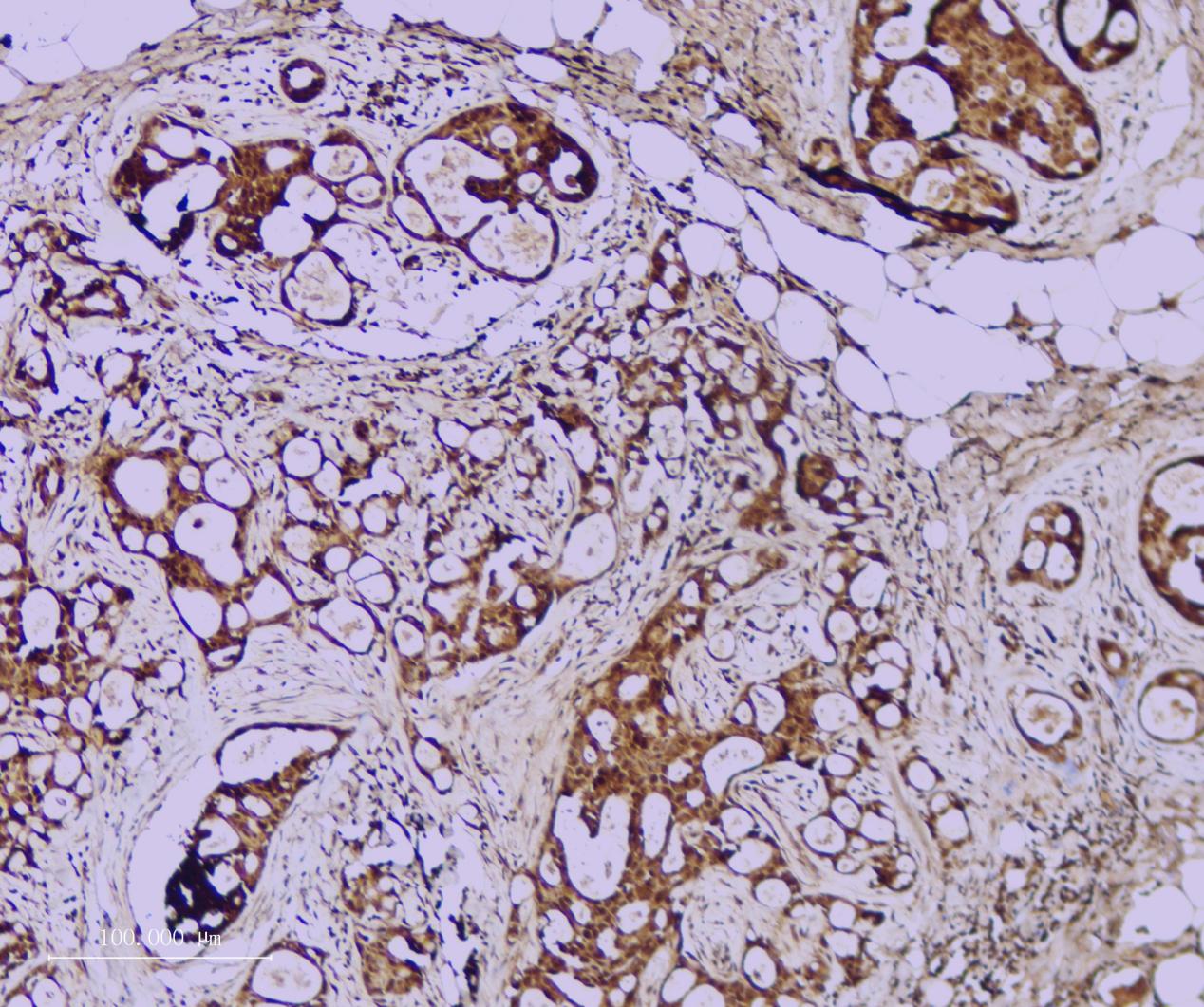

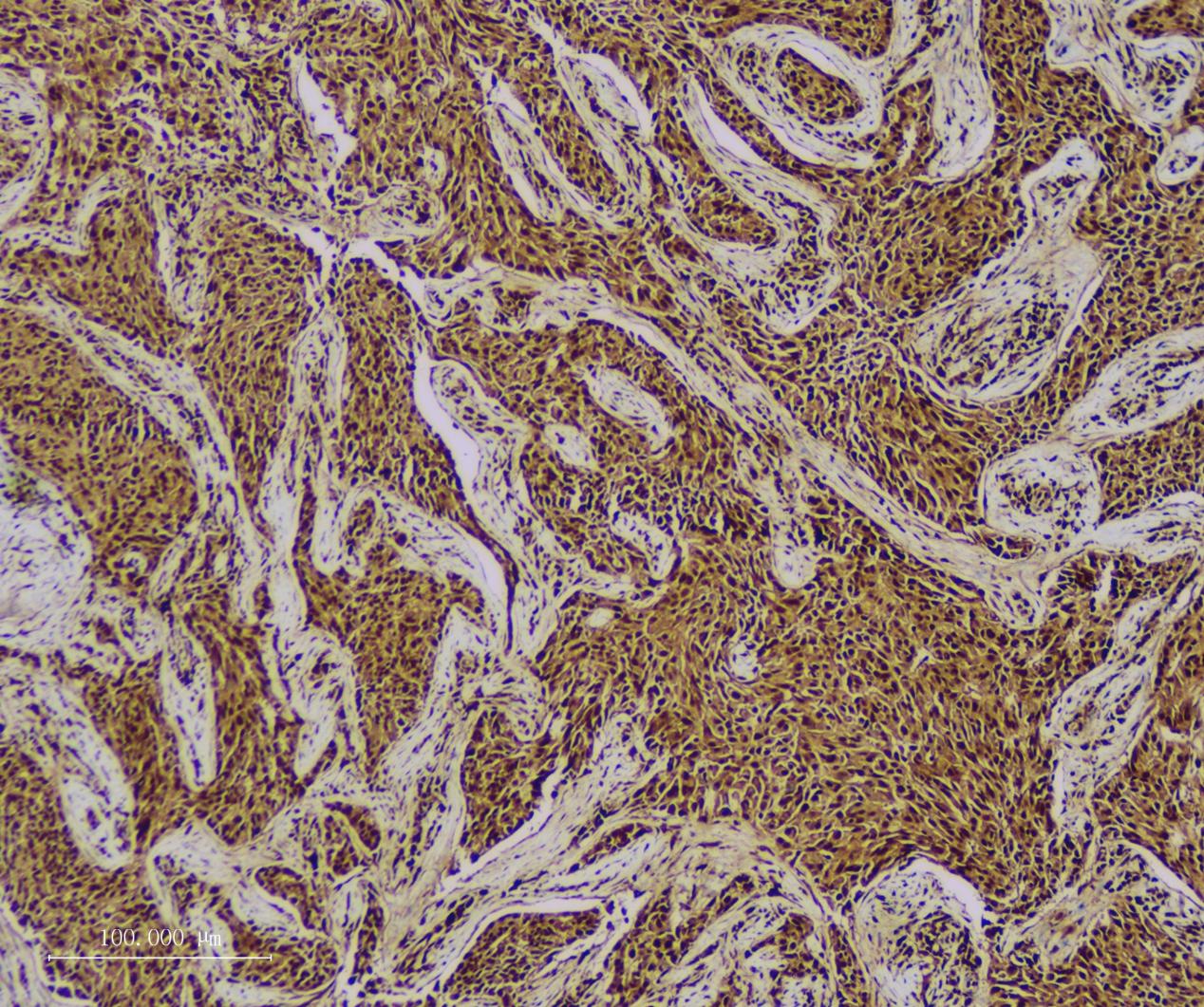

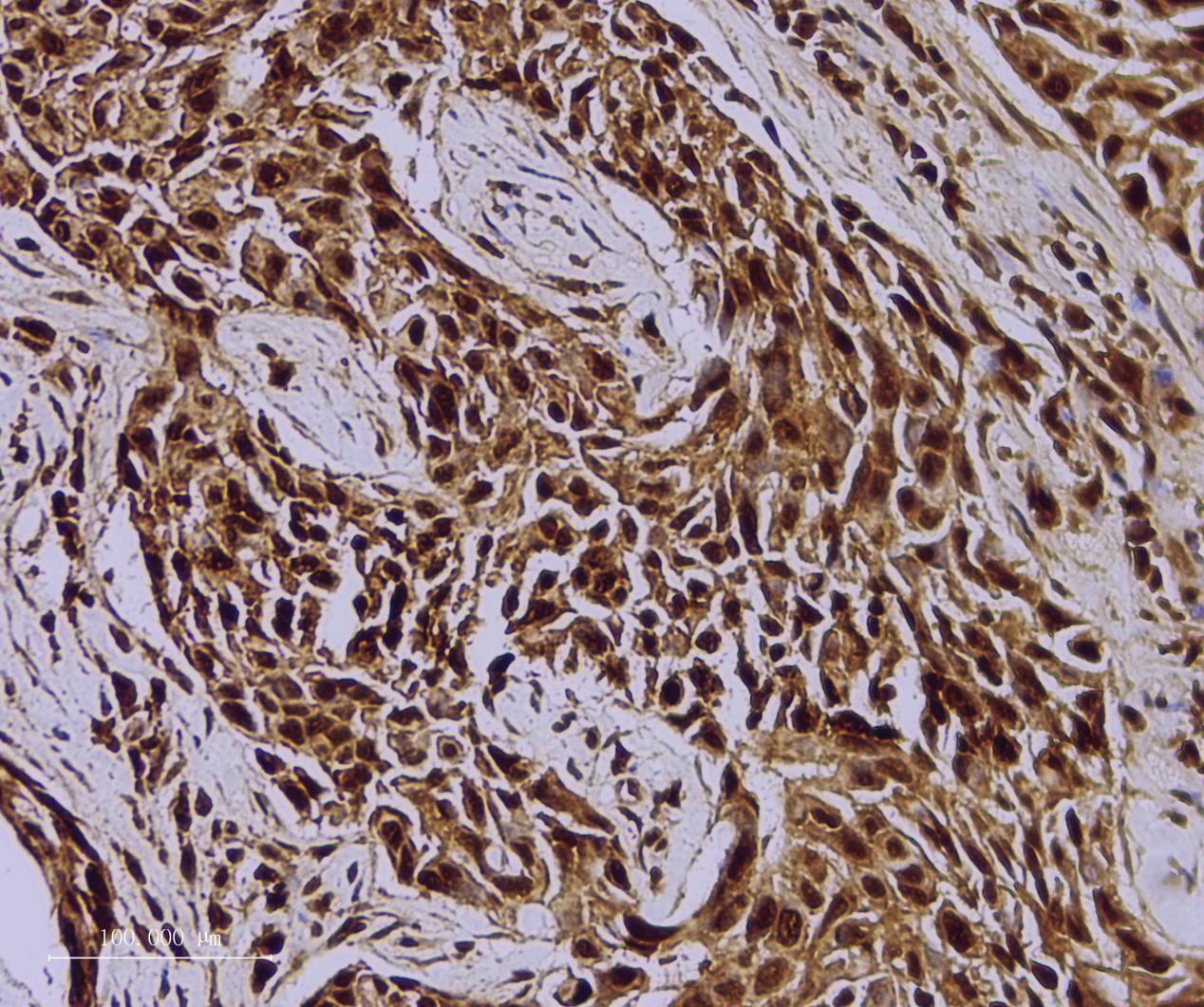

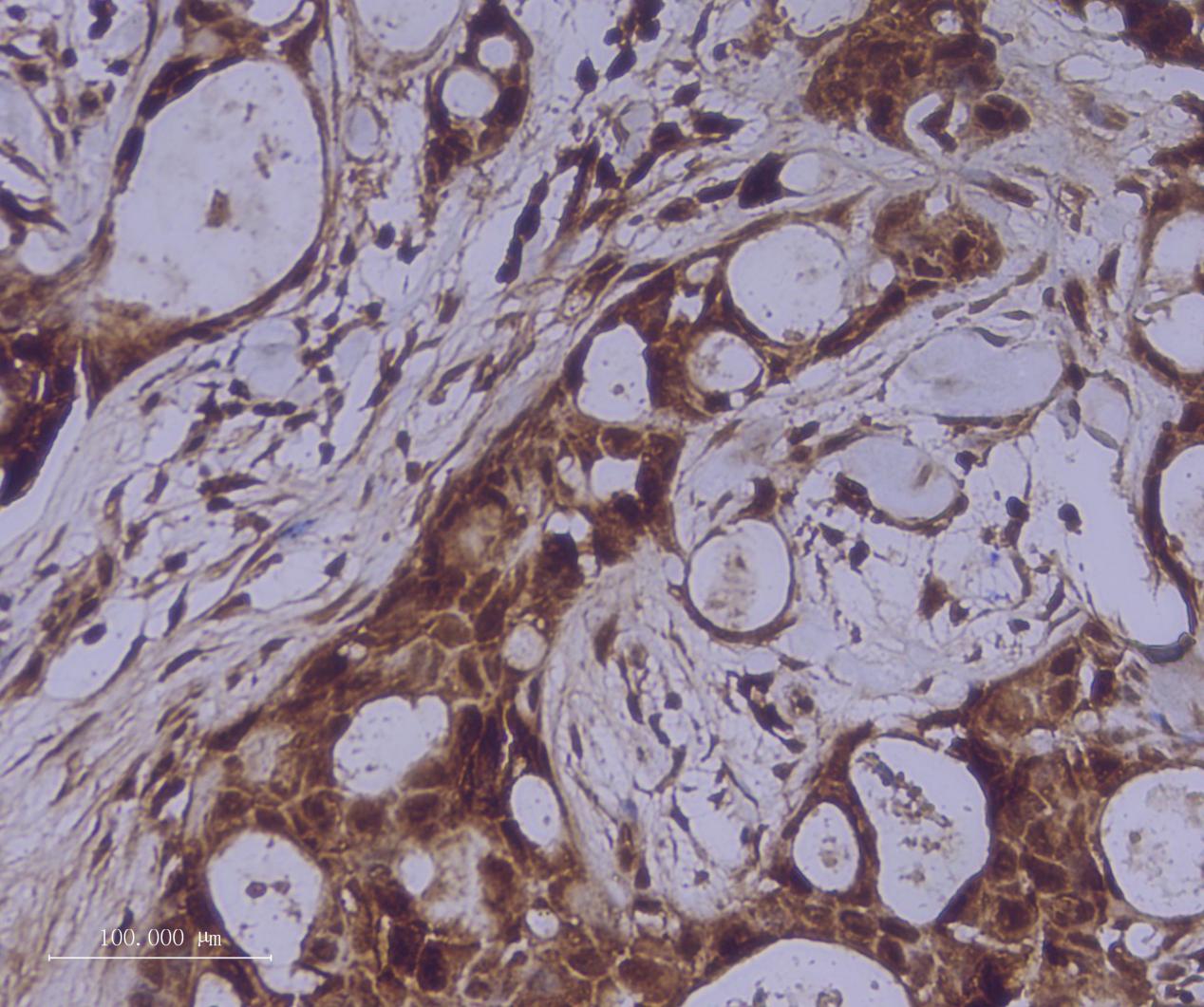

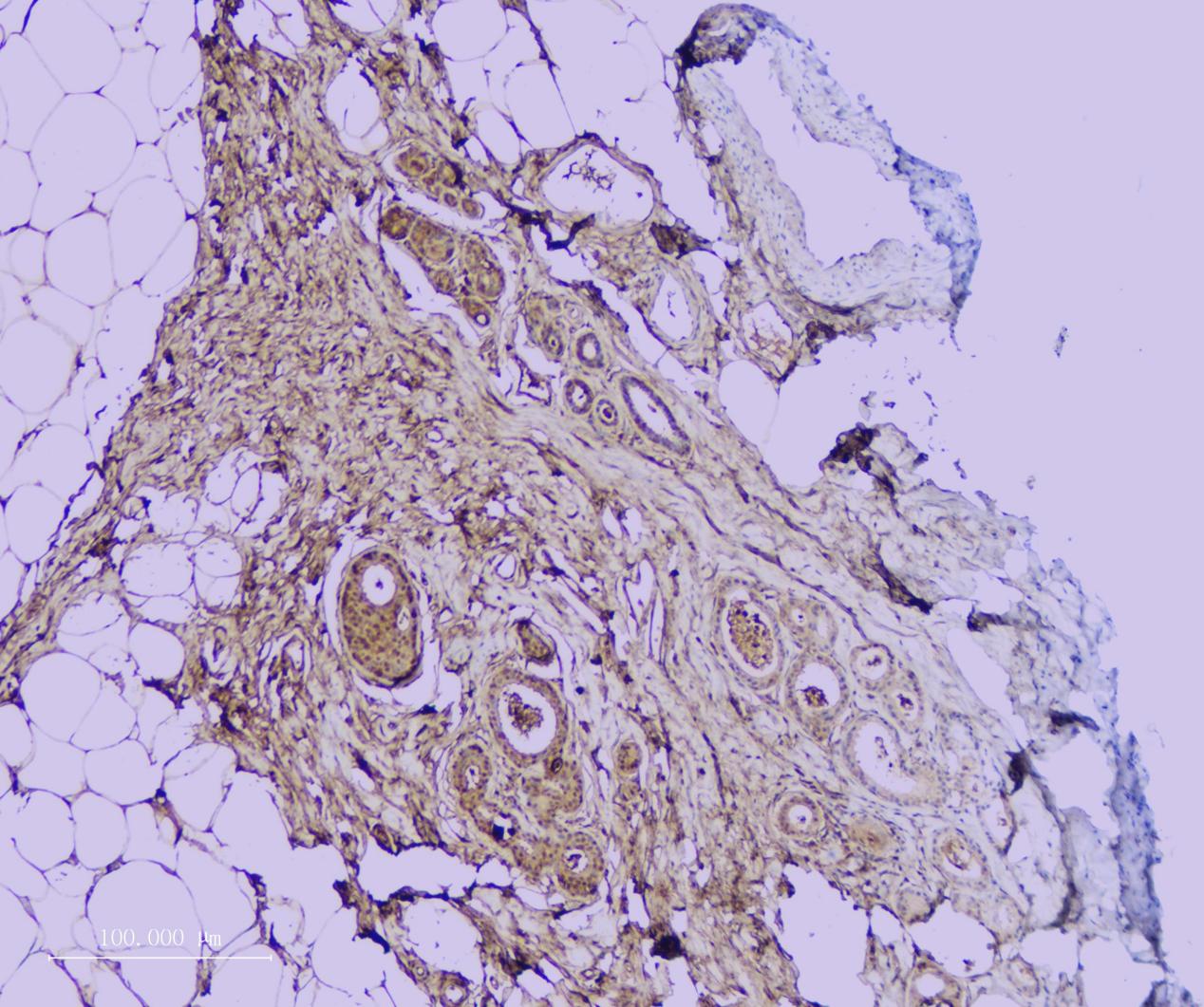

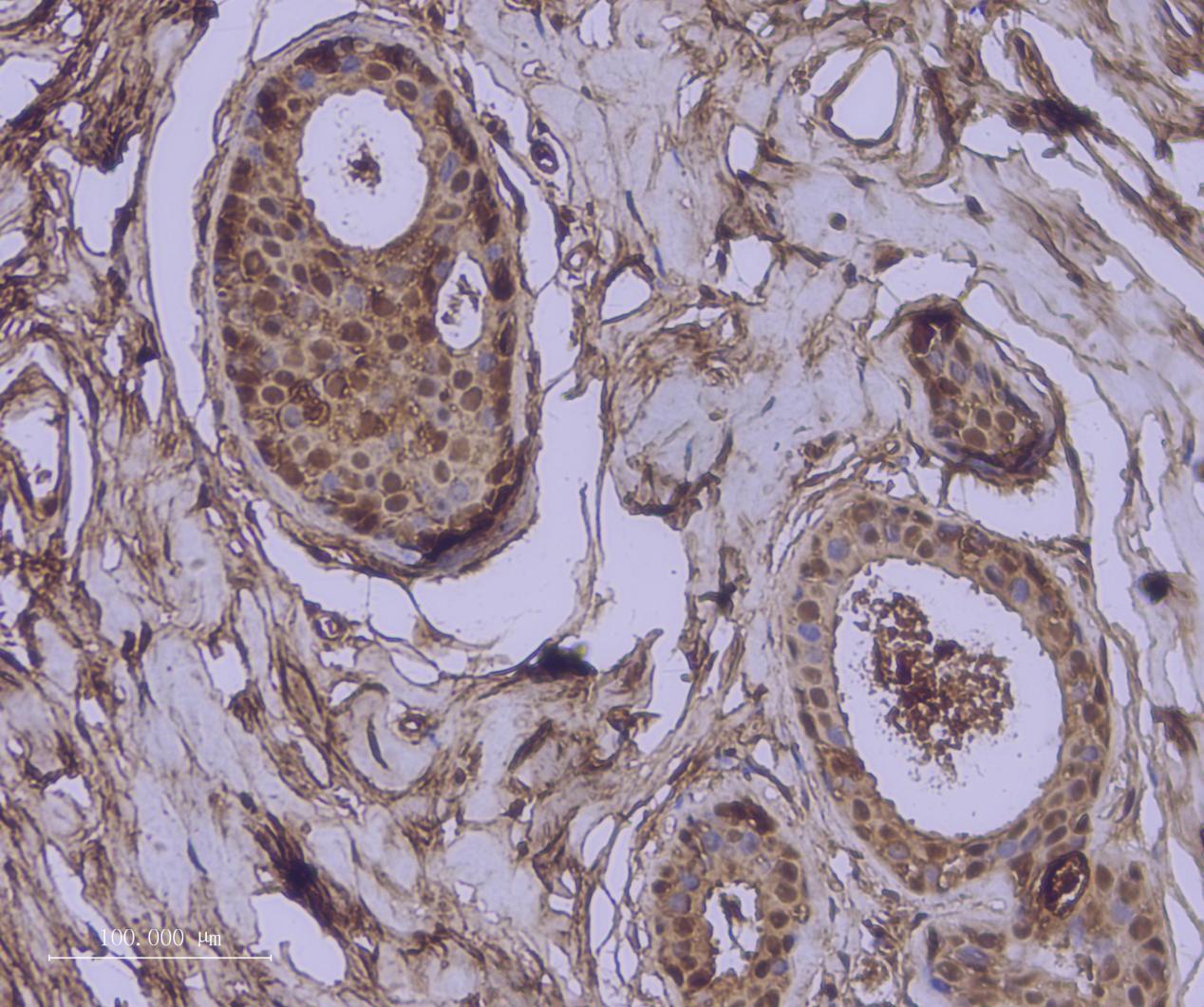

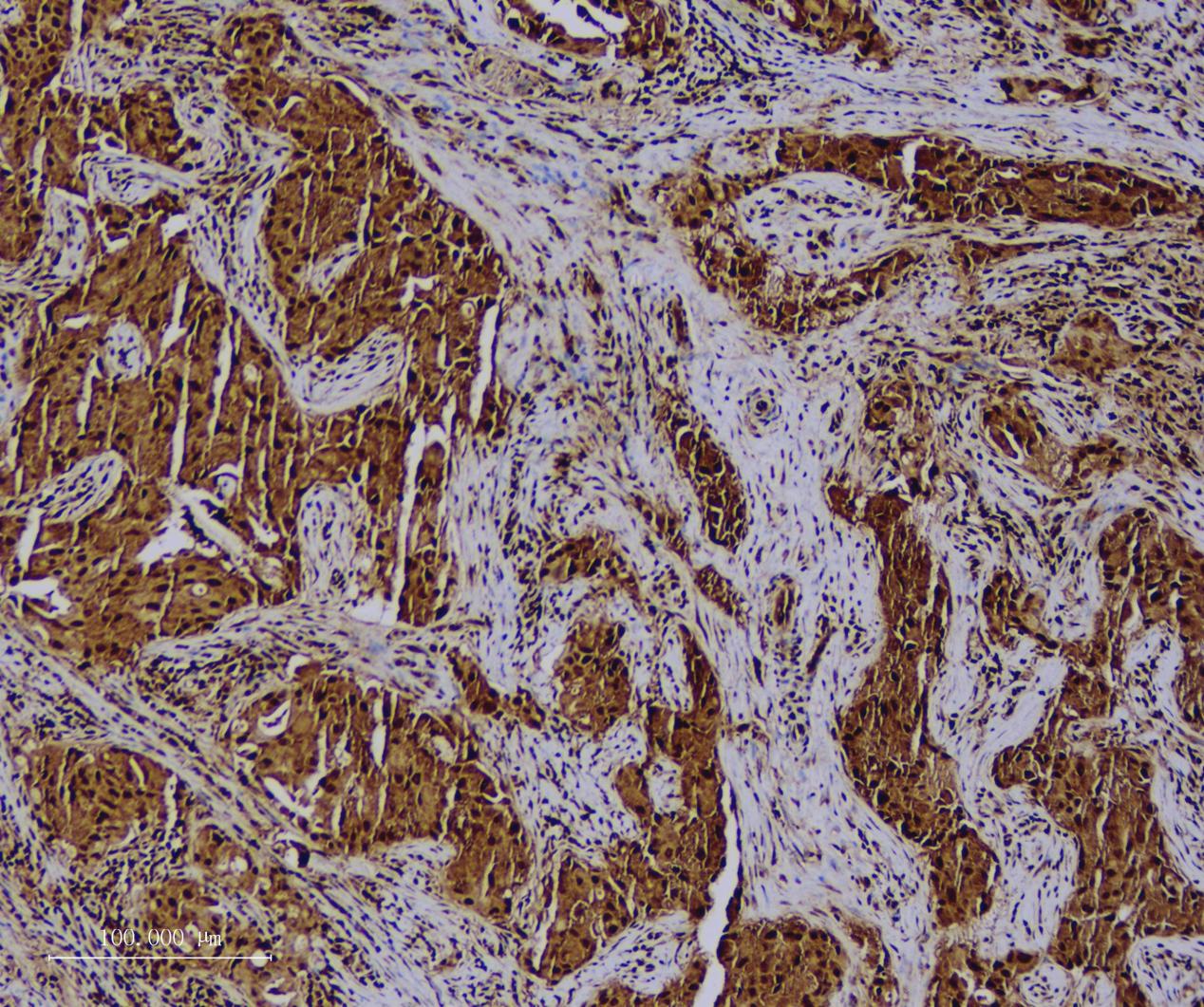

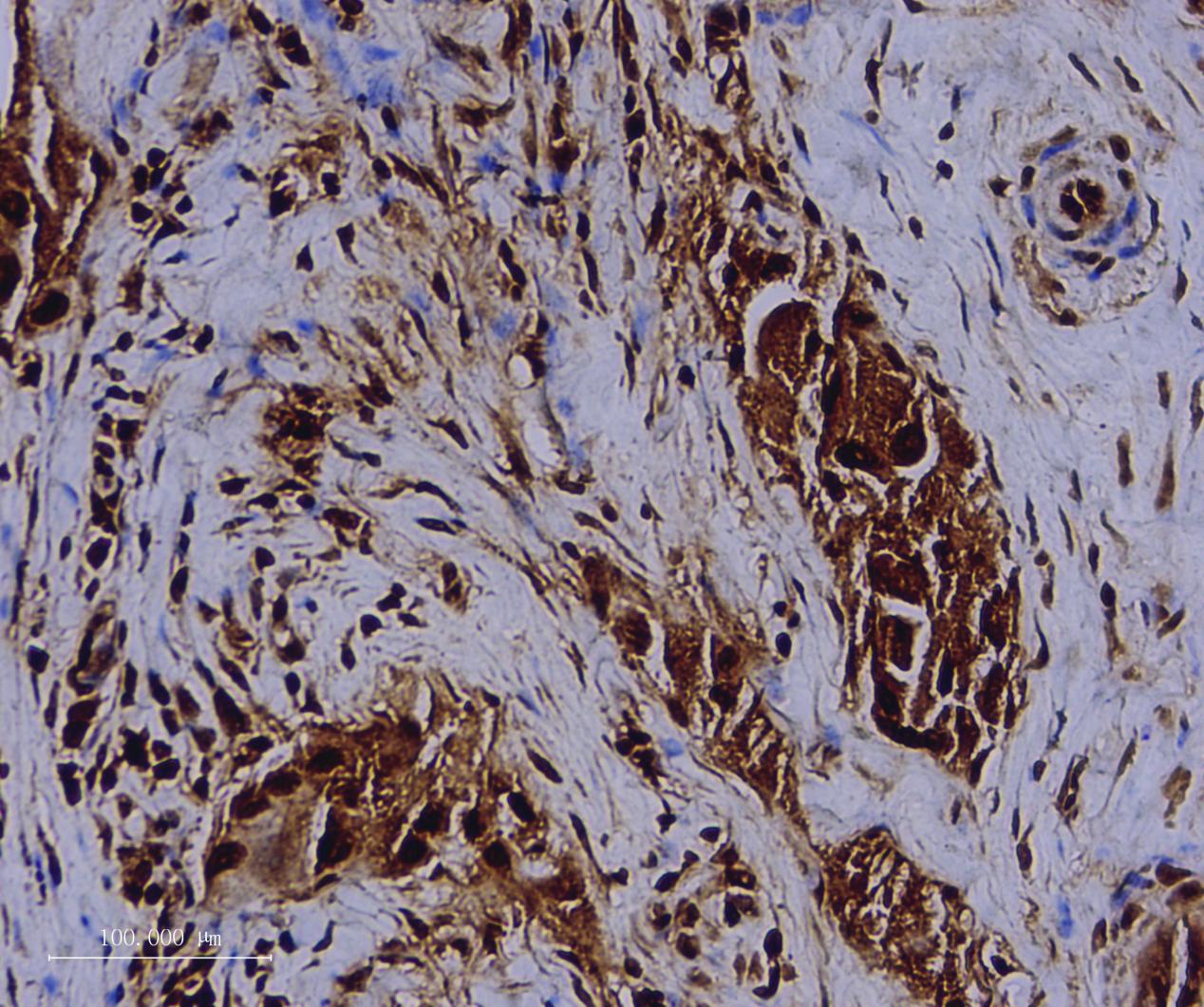

Supplement: S1 File — (DOCX) [file pone.0341357.s008.docx]

Figure_8E_MDA-MB-231_siCtrl


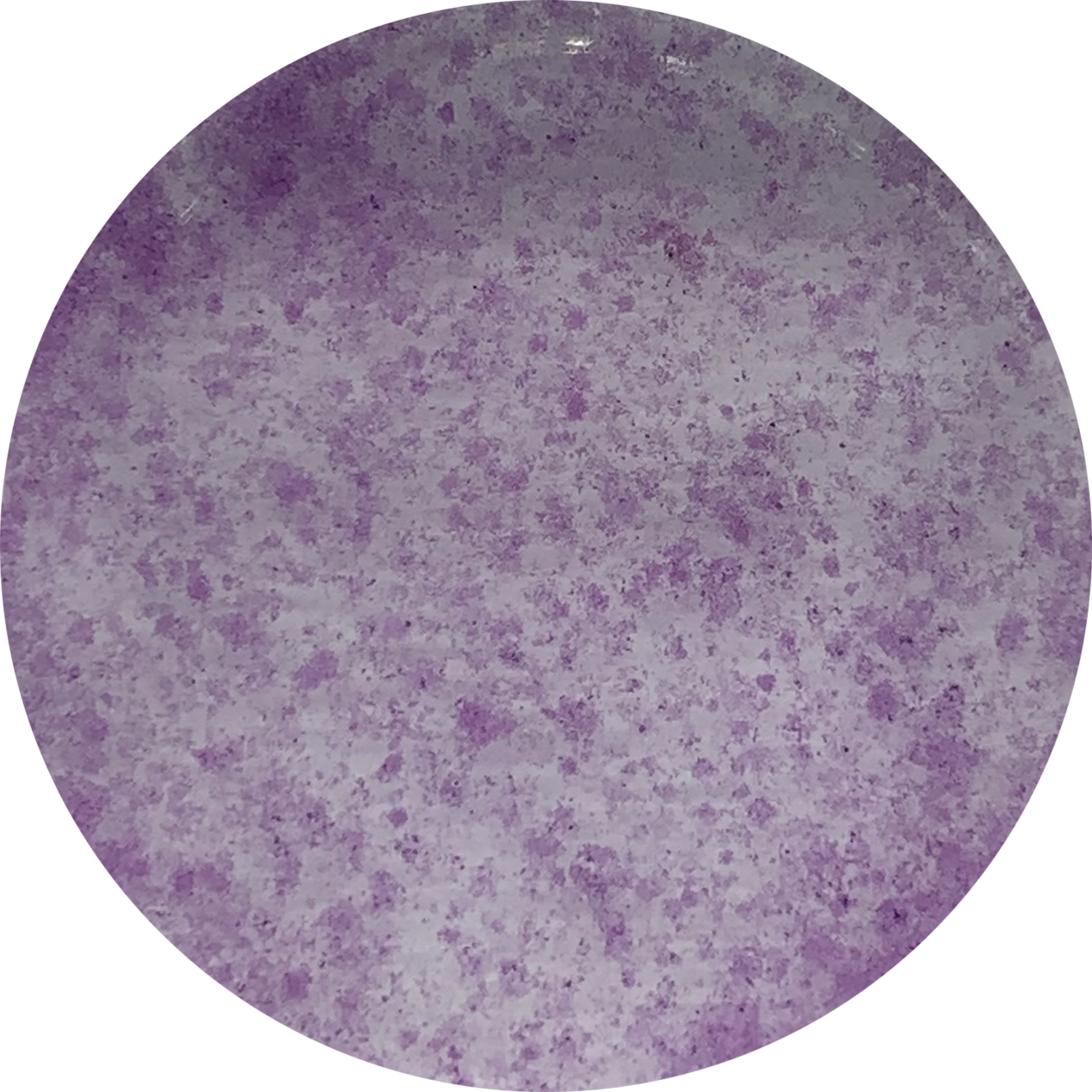


Figure_8E_MDA-MB-231_siRUBCN#1


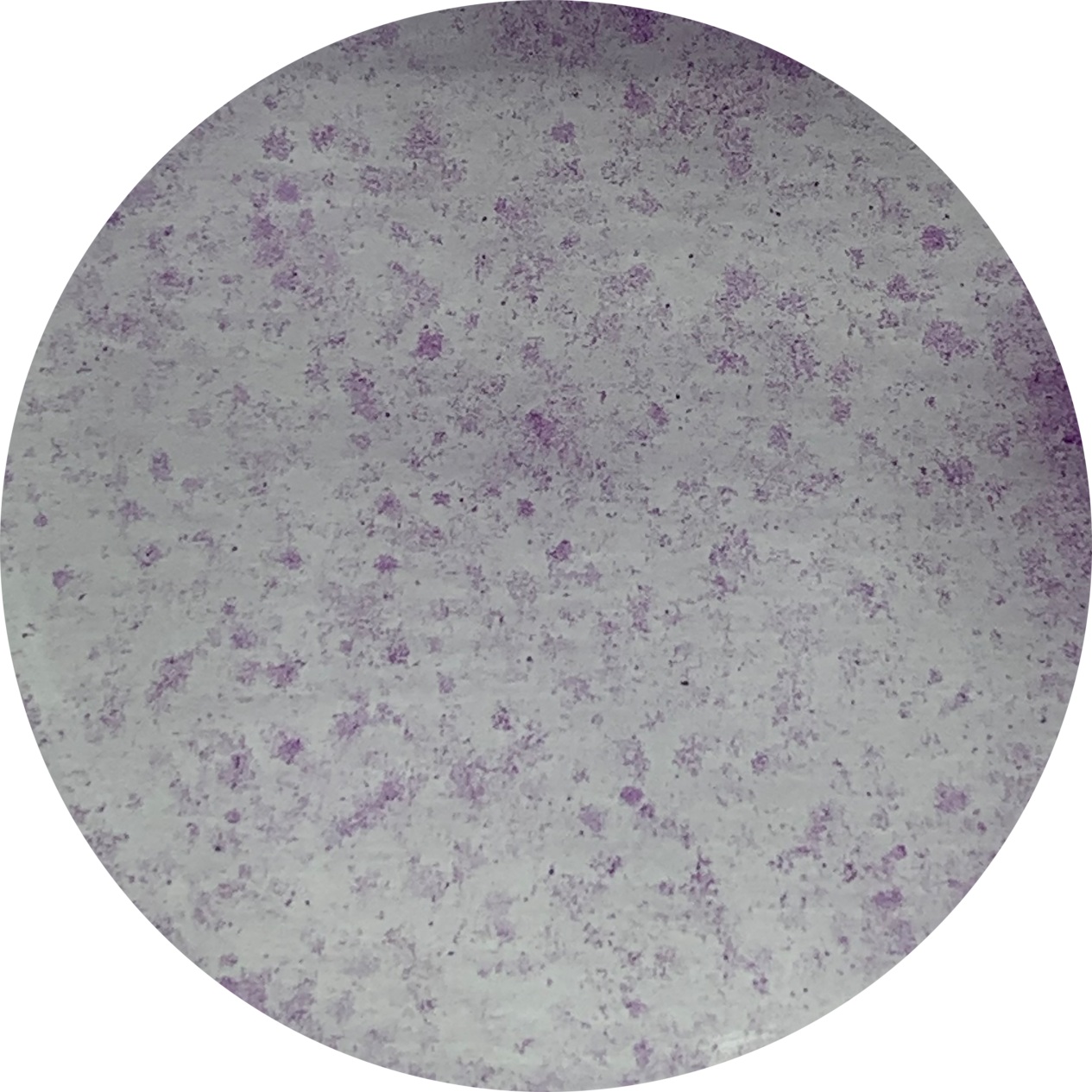


Figure_8E_MDA-MB-231_siRUBCN#2


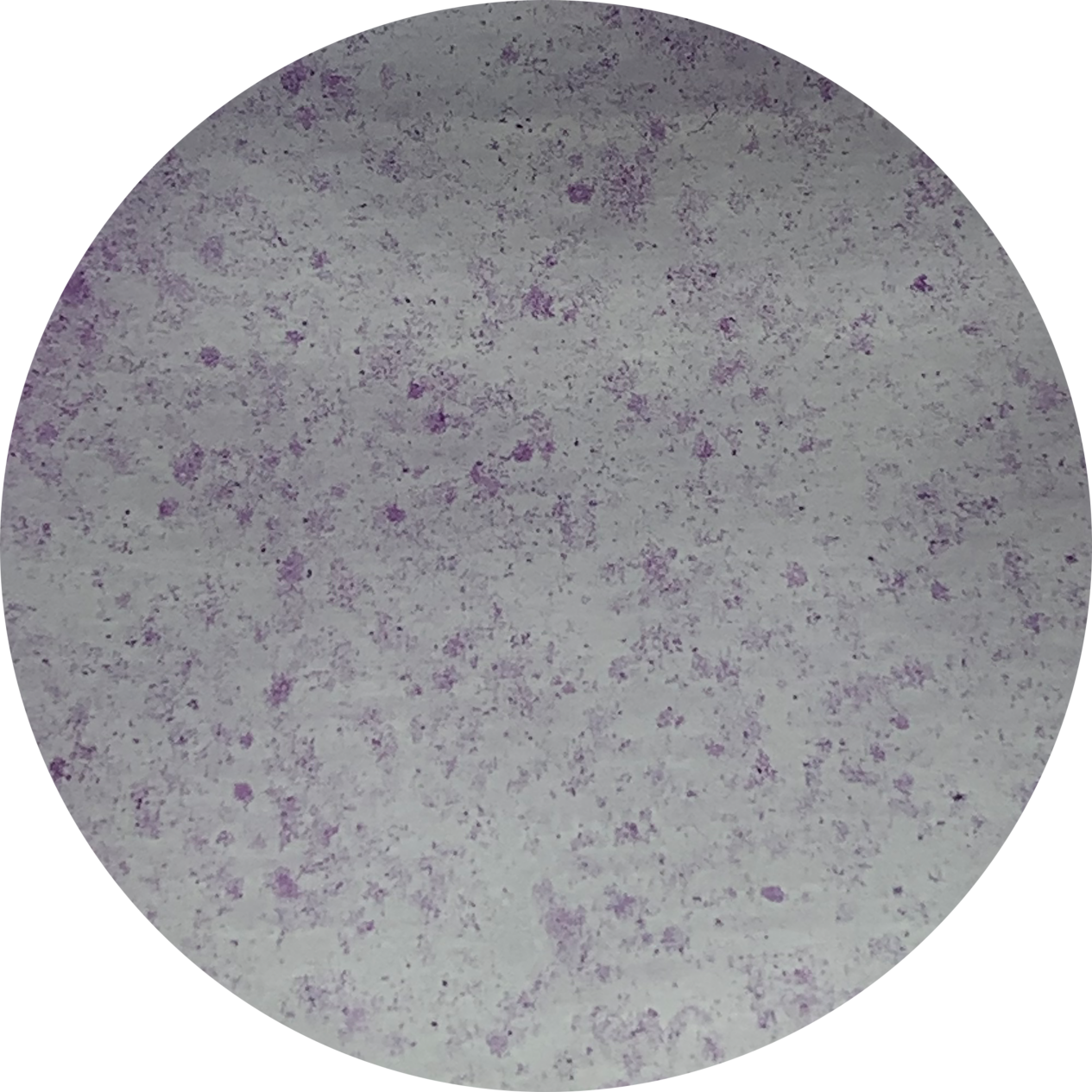

Supplement: S2 File — (DOCX) [file pone.0341357.s009.docx]

Figure_8G_MDA-MB-231_siCtrl
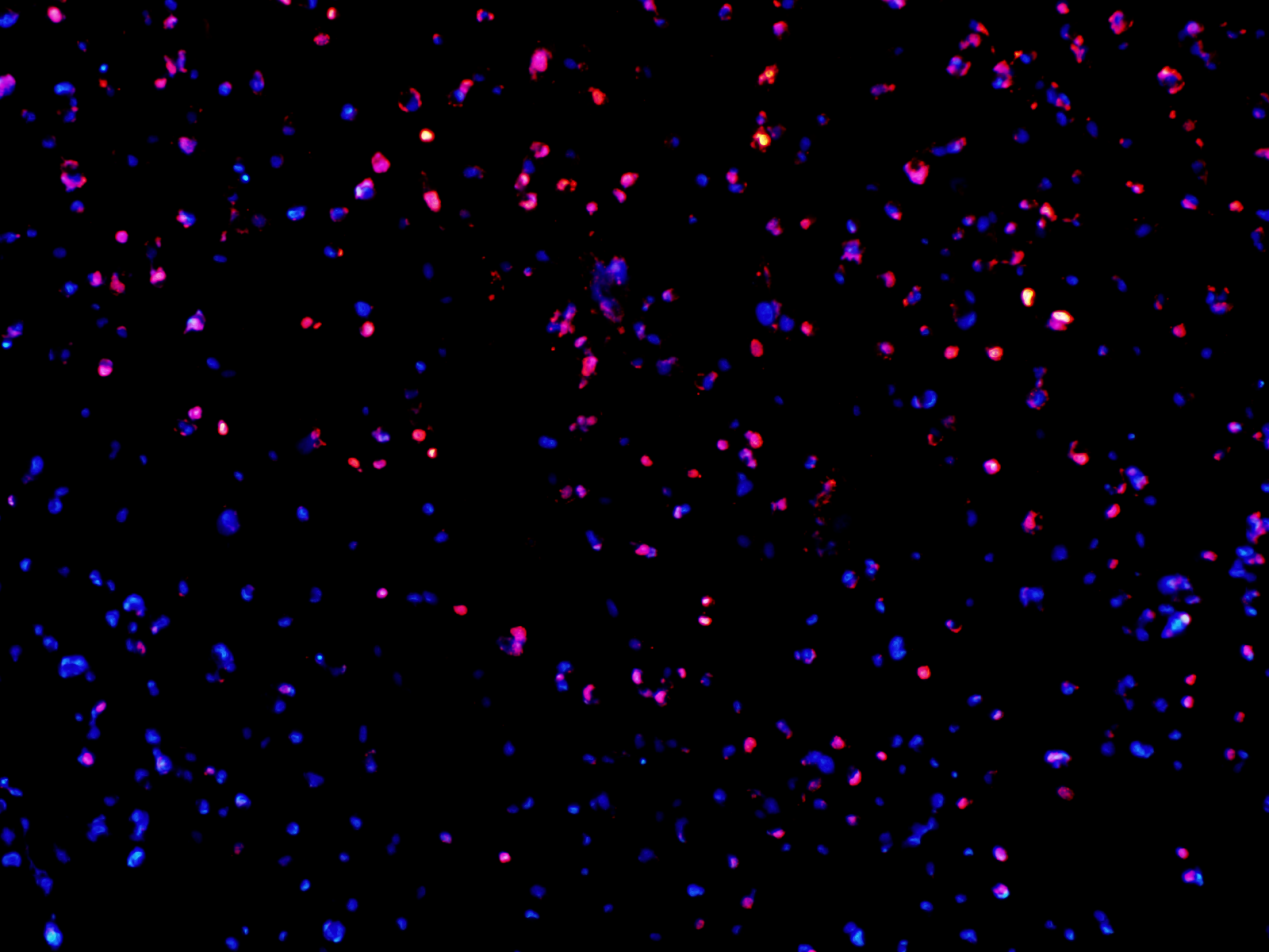

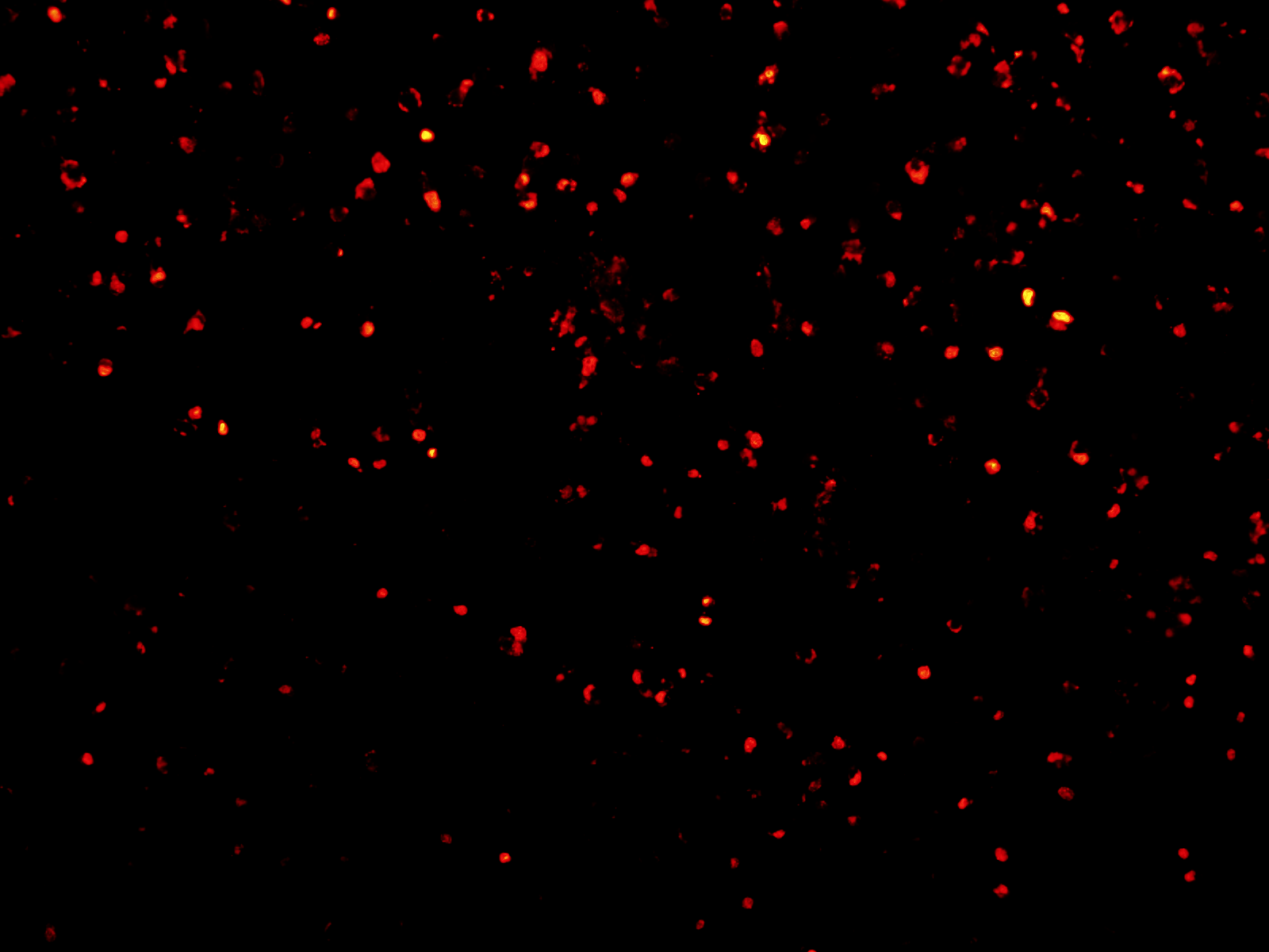

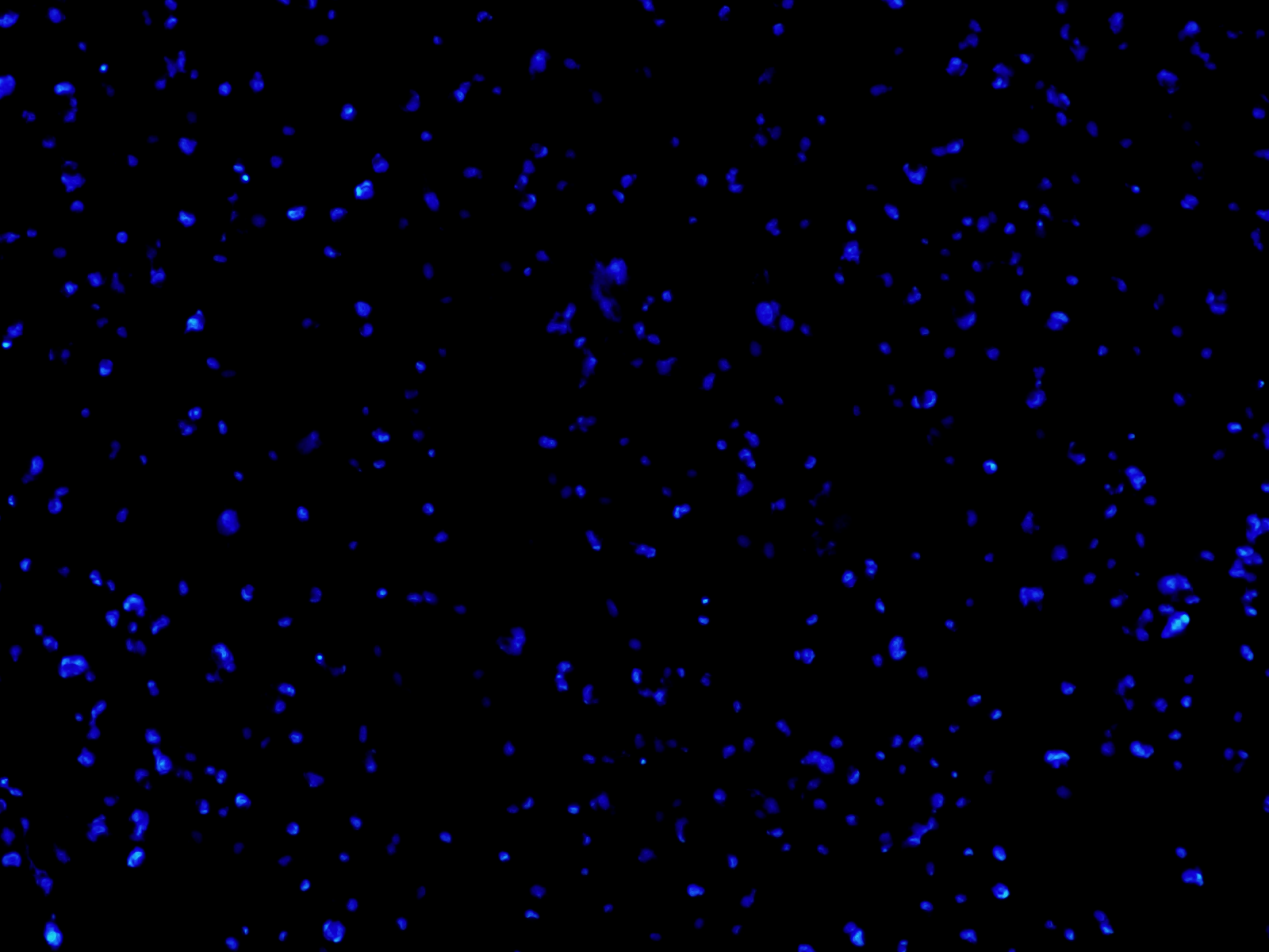
Figure_8G_MDA-MB-231_siRUBCN#1


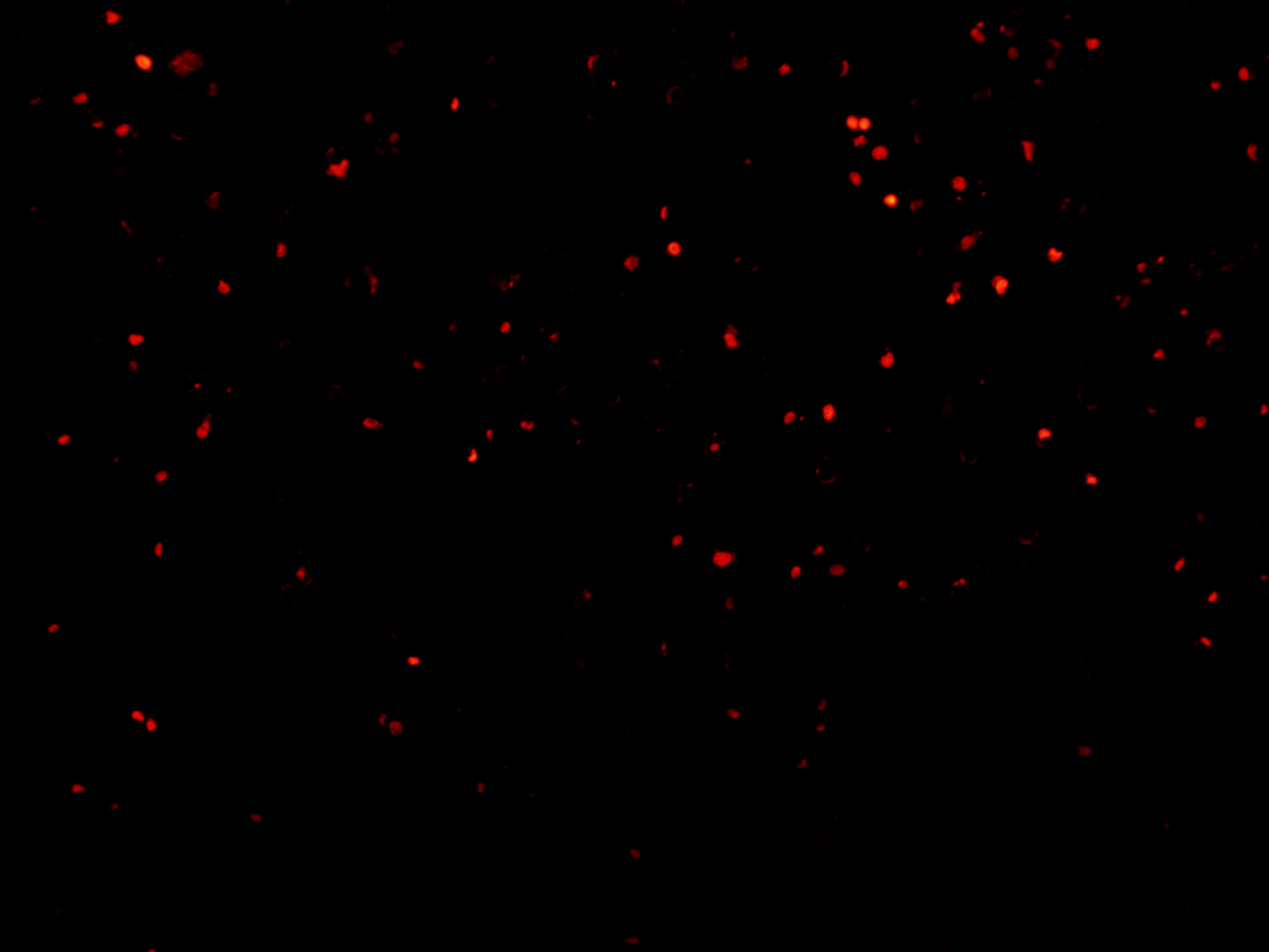

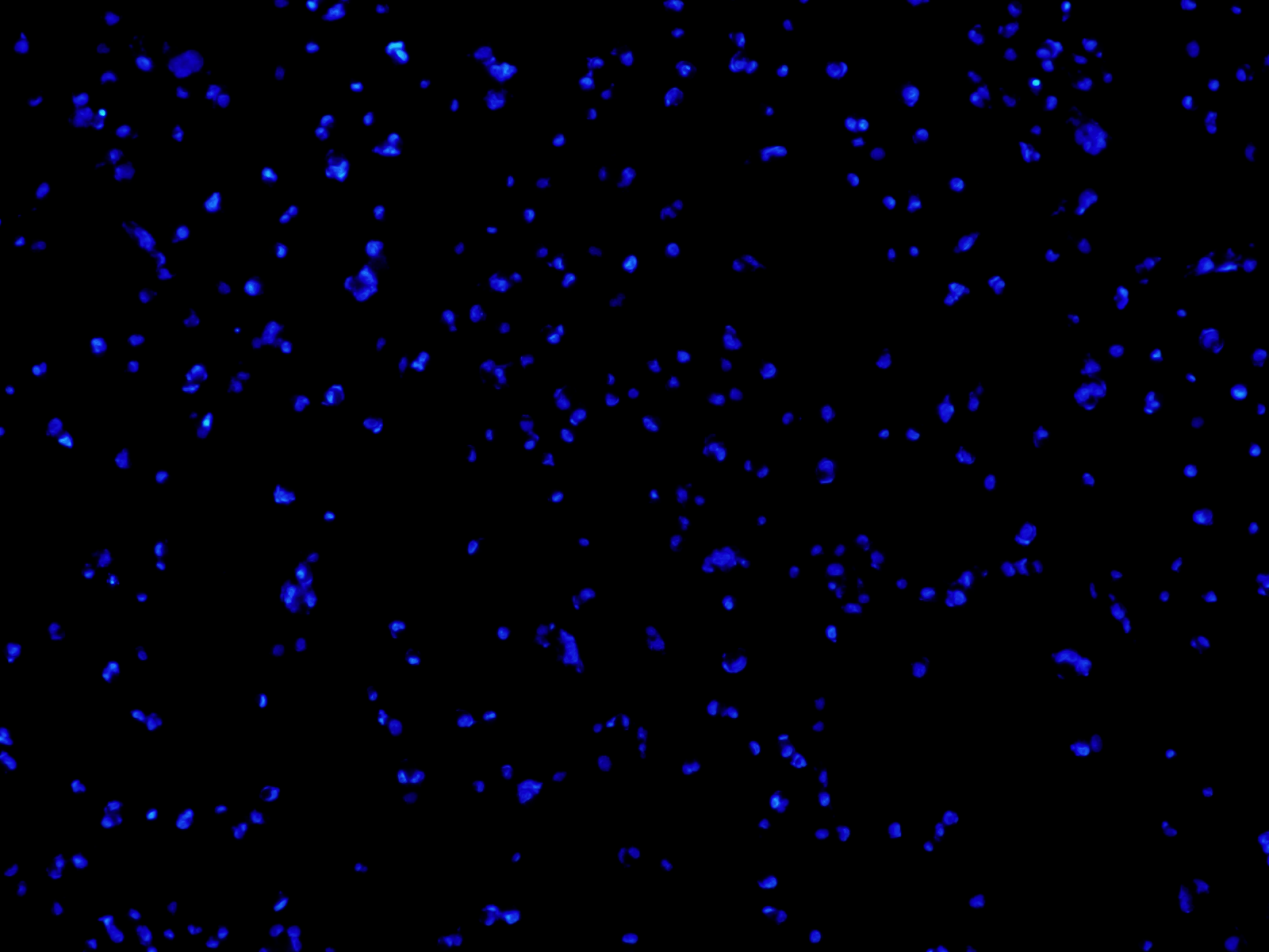

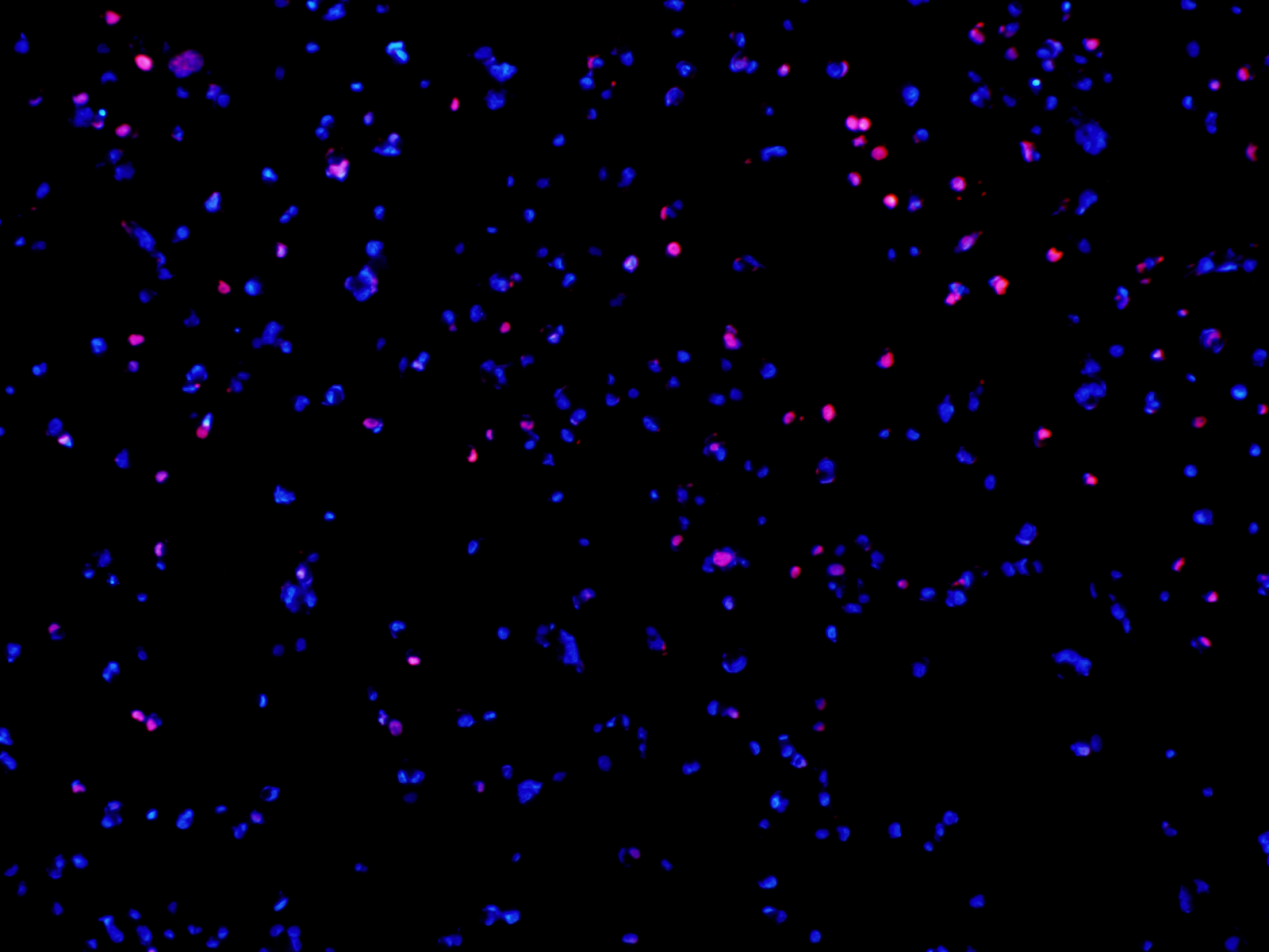


Figure_8G_MDA-MB-231_siRUBCN#2


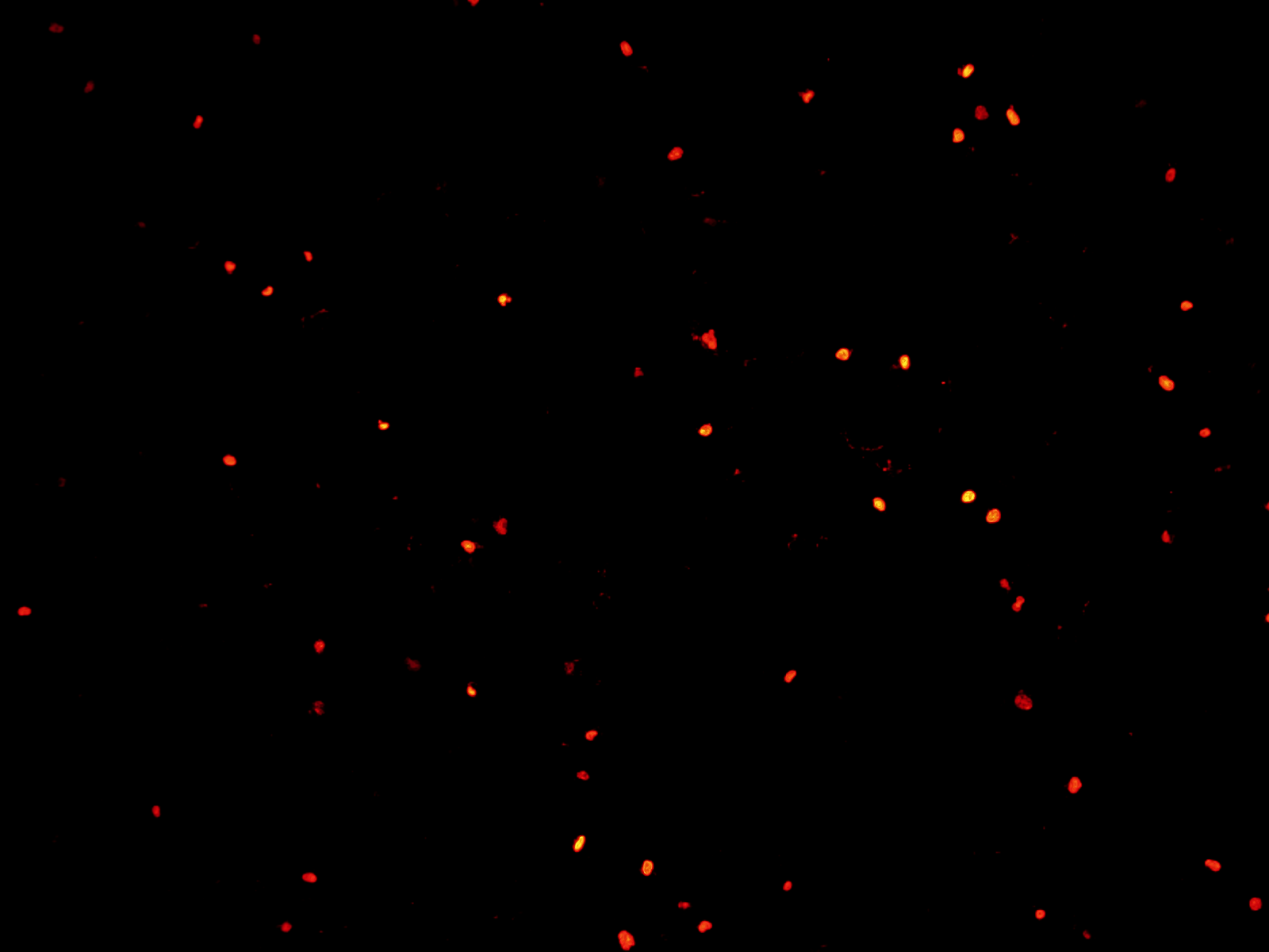

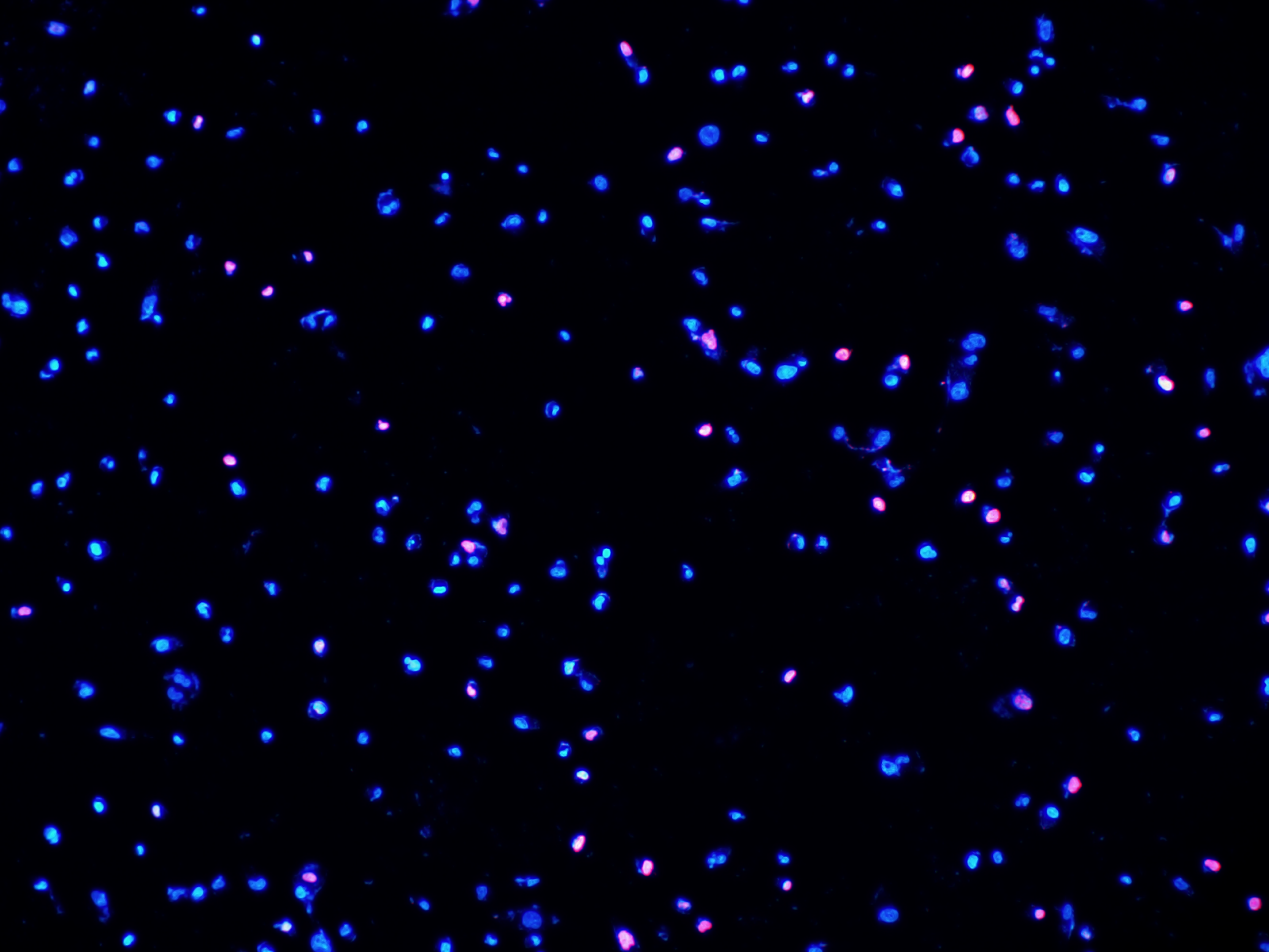

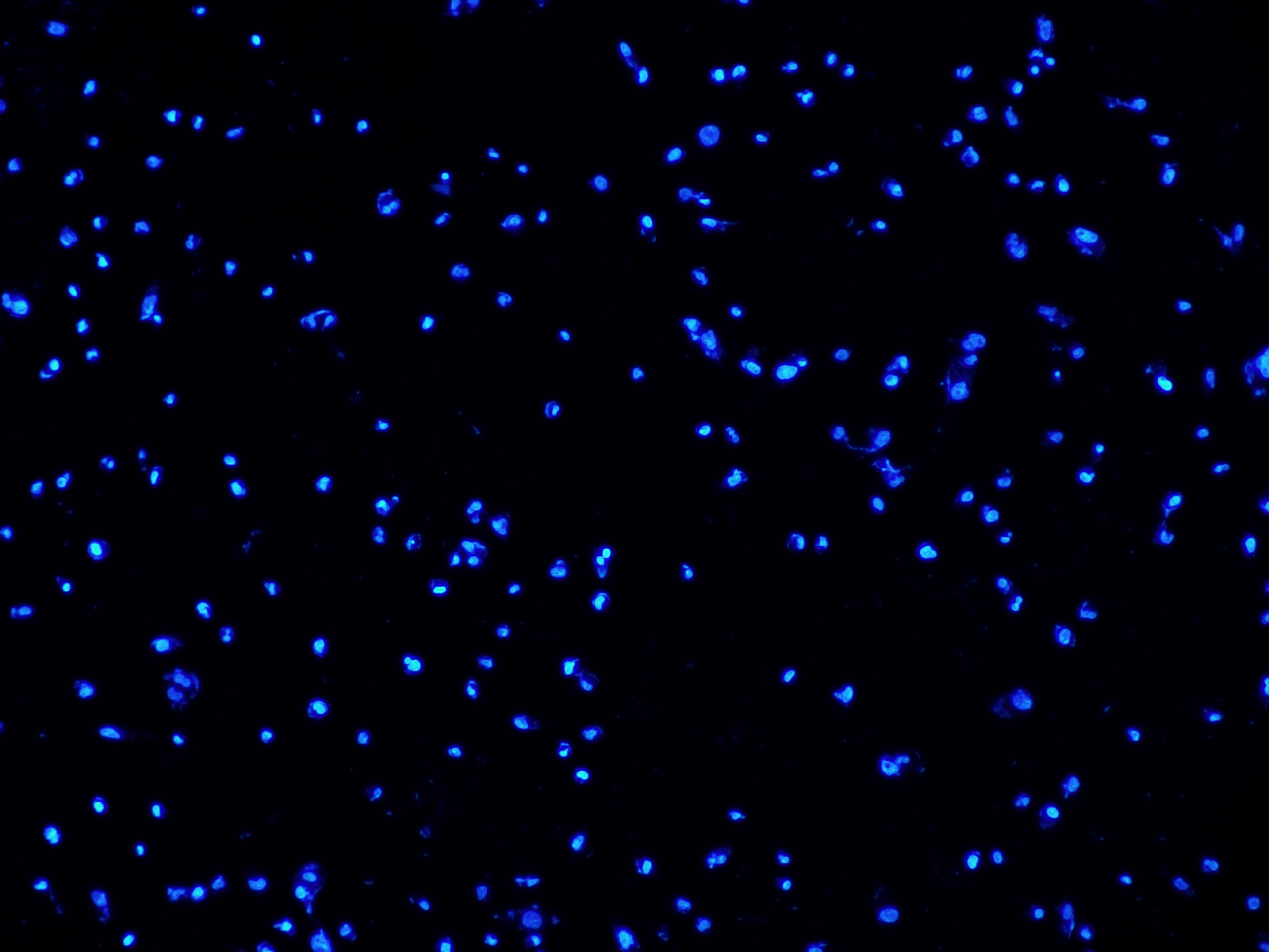

Supplement: S4 File — (DOCX) [file pone.0341357.s011.docx]

Figure_9B_MDA-MB-231_siCtrl
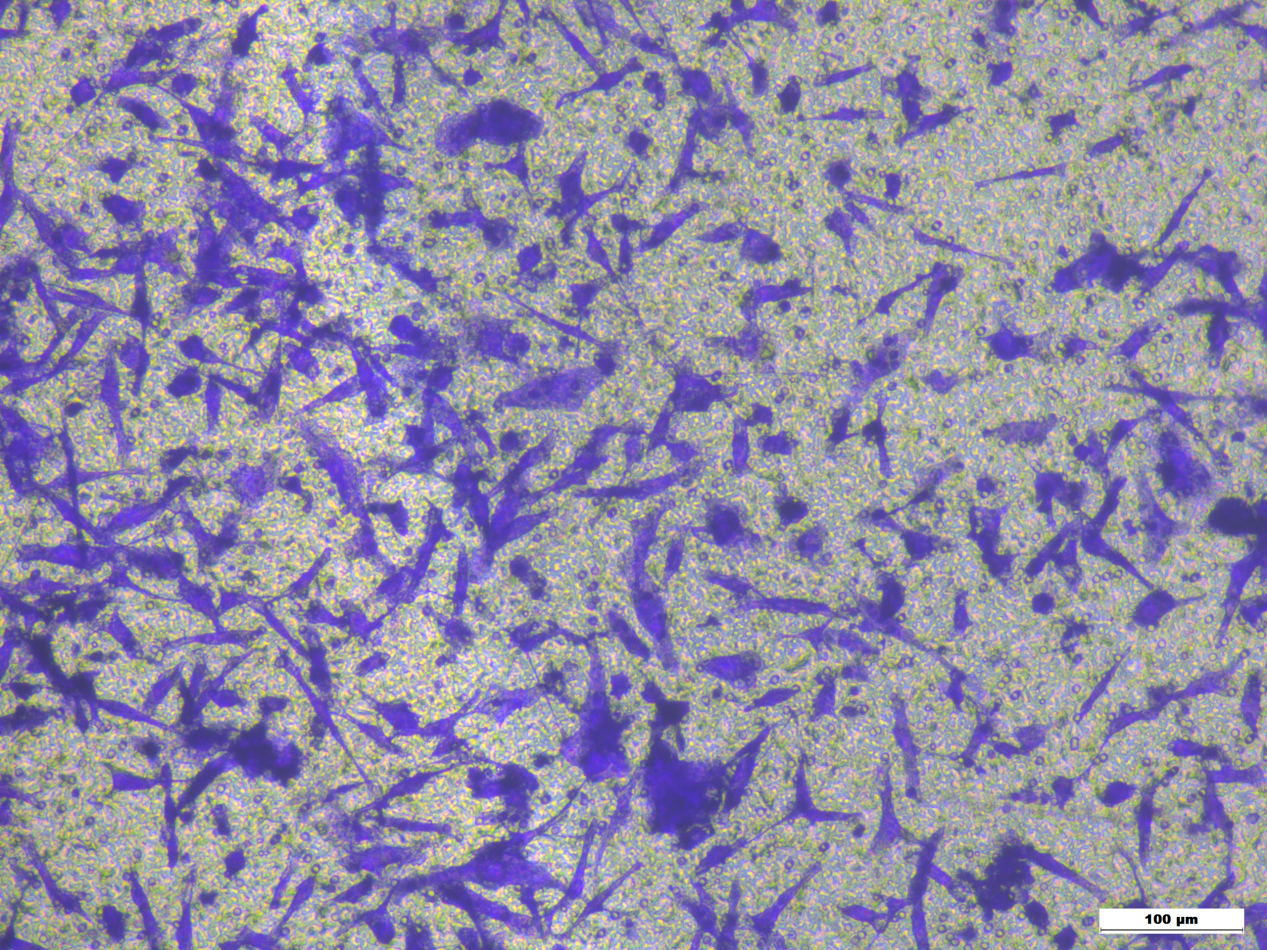


Figure_9B_MDA-MB-231_siRUBCN#1


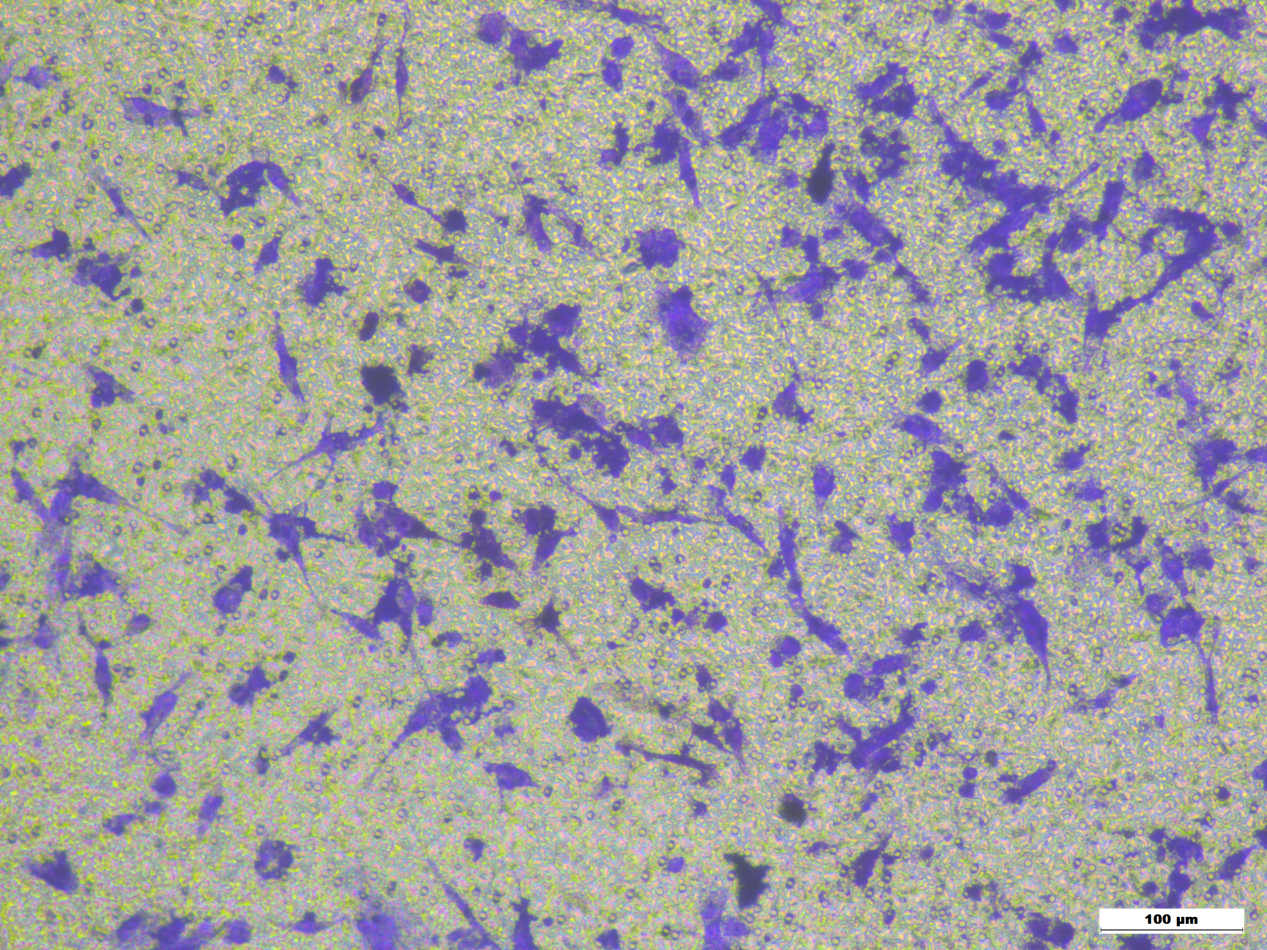
Figure_9B_MDA-MB-231_siRUBCN#2


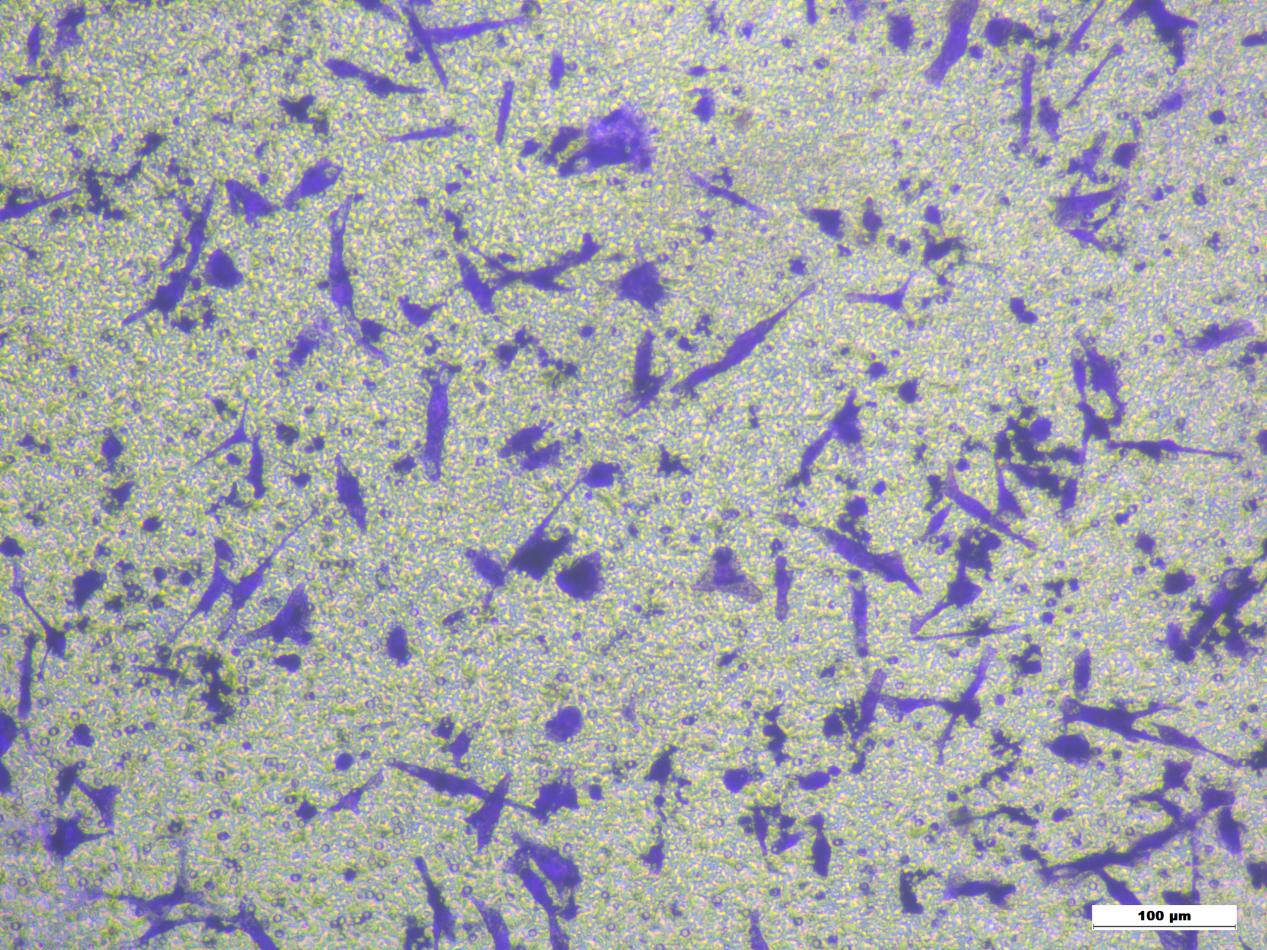

Supplement: S7 File — (DOCX) [file pone.0341357.s014.docx]

Figure_9A_MDA-MB-231_siCtrl (0h,24h)
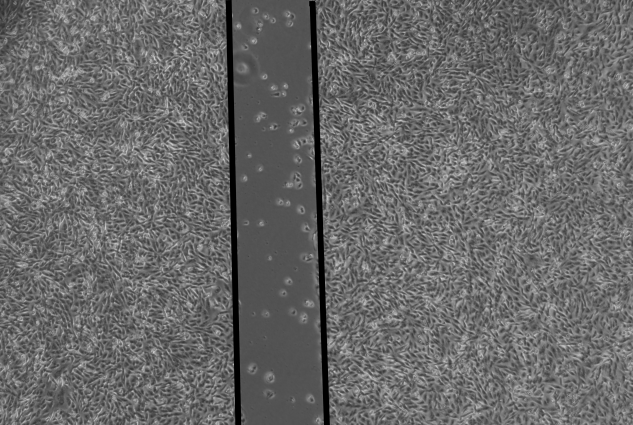

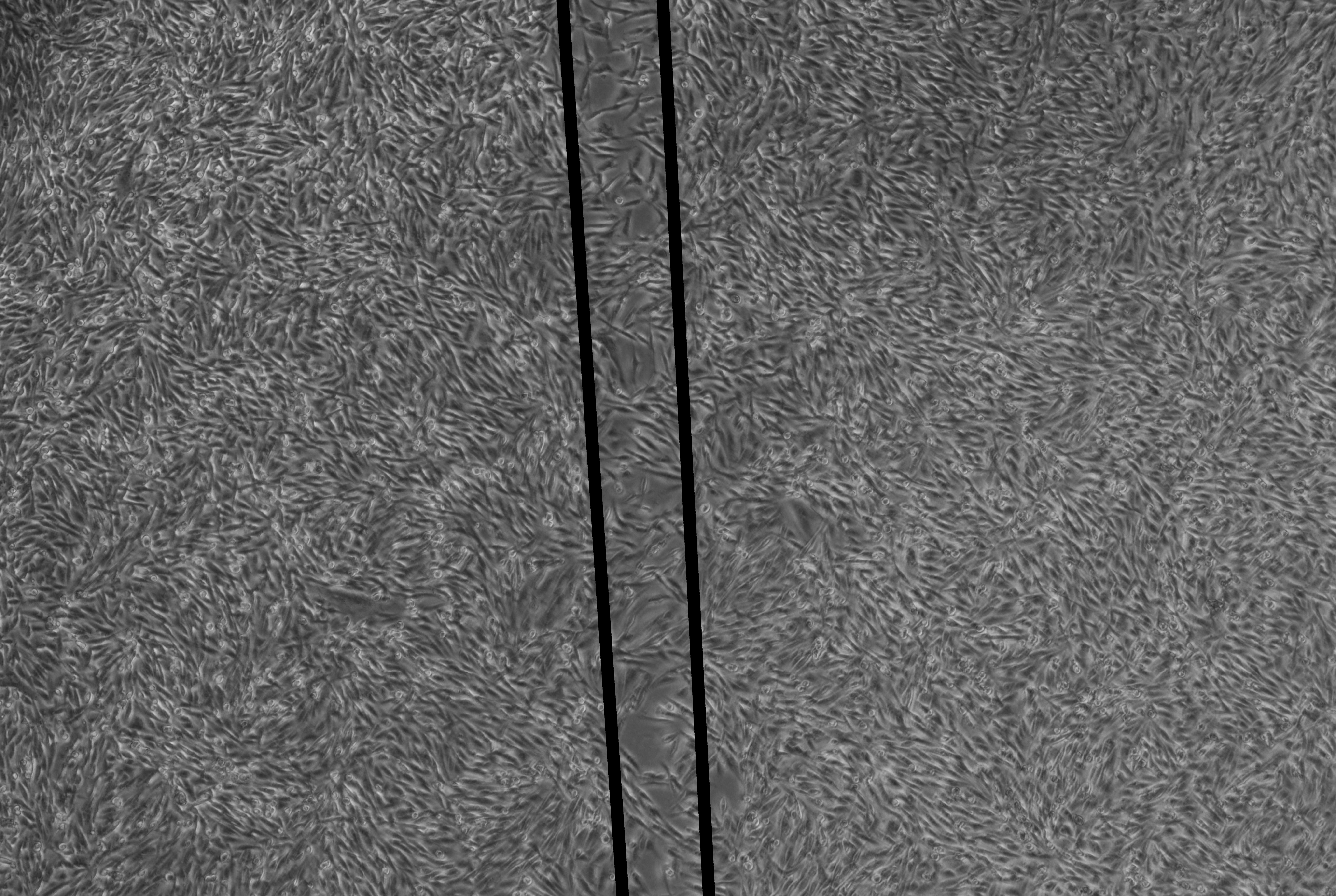


Figure_9A_MDA-MB-231_siRUBCN#1 (0h,24h)
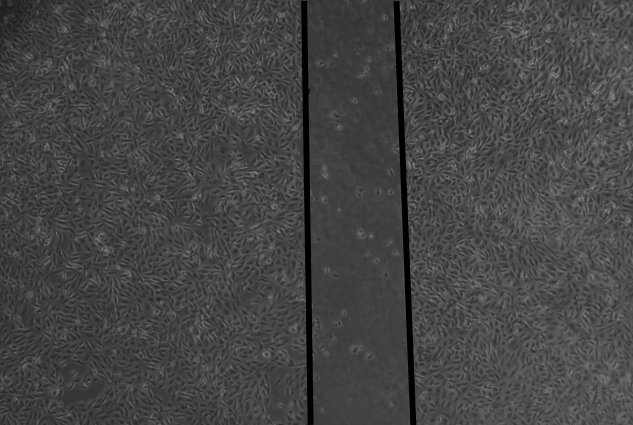

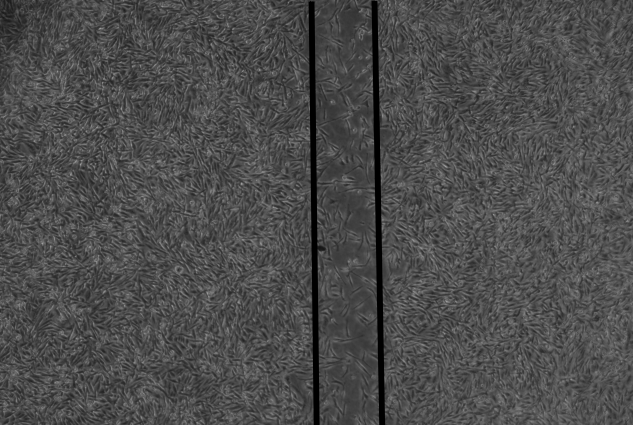


Figure_9A_MDA-MB-231_siRUBCN#2 (0h,24h)


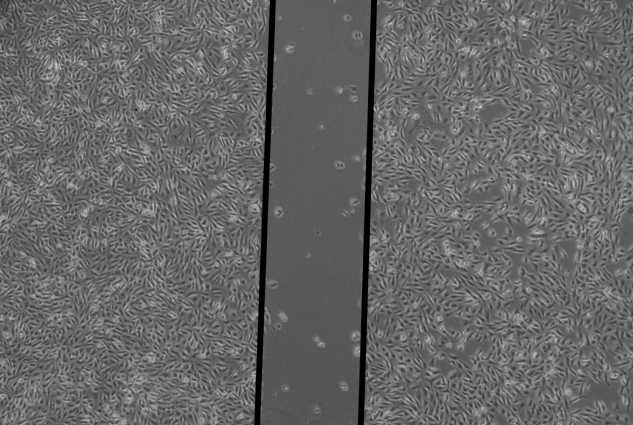

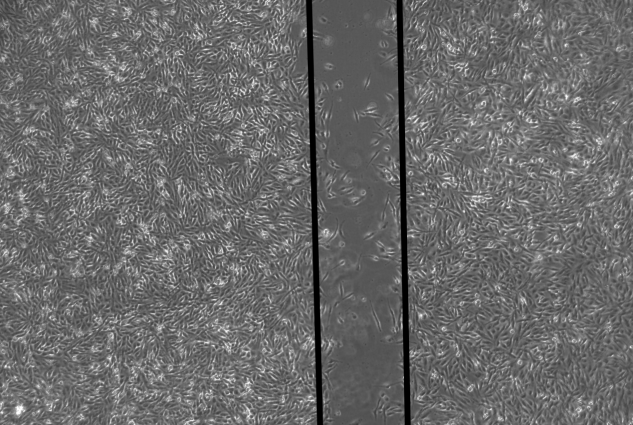

Supplement: S8 File — (DOCX) [file pone.0341357.s015.docx]
