# Supplementary material for: RUBCN as a novel prognostic biomarker and therapeutic target in breast cancer
Source: PLoS One. 2026 Jan 27;21(1):e0341357. doi: 10.1371/journal.pone.0341357 (PMC12843558; doi:10.1371/journal.pone.0341357)
Supplement: Attachment — Submitted filename: Response to Reviewers.docx [file pone.0341357.s017.docx]

Title of the manuscript: RUBCN as a Novel Prognostic Biomarker and Therapeutic Target in Breast Cancer

Manuscript Number: PONE-D-25-46002

Dear Dr. Amy McCart Reed and Reviewers,

We wish to express our sincere gratitude for the opportunity to revise our manuscript and for your consideration of it. We greatly appreciate the time and effort invested by the editor and the reviewers. Their insightful and constructive comments have been invaluable in helping us significantly improve the quality and rigor of our work.

We have carefully addressed all the points raised in the reviewers' comments. Our point-by-point responses are detailed below, where we have outlined all the modifications made. The corresponding changes have been incorporated into the revised manuscript and are highlighted using the "Track Changes" feature.

Part 1: Response to Journal Requirements

We have thoroughly reviewed and ensured that our manuscript adheres to all formatting guidelines specified by PLOS ONE. The following items have been addressed:

1. Format template: The manuscript has been revised in accordance with the PLOS ONE formatting template provided in the link.

2. Code sharing: The code used in this study has been uploaded to GitHub. The relevant information has been provided in the Data Availability Statement.

3. Funding Statement: Regarding the funder statement, we have updated the corresponding section in the Cover Letter as follows: "The funders had no role in the study design, data collection and analysis, decision to publish, or preparation of the manuscript."

4. Western Blot Data: We have prepared the full, uncropped original images for all Western blot experiments.

5. References: We have verified all references to ensure they are complete, accurate, and that no retracted papers have been cited.

Reviewer #1:

Comment 1: The narrative flow of the Introduction section could be enhanced. For instance, the description of the RCD pathway and the dual role of autophagy could be clearer, and the transition from ATG4A to RUBCN is somewhat abrupt.

Author's reply: We thank the reviewer for this valuable suggestion. Accordingly, we have rewritten the Introduction section to enhance its logical flow.

Location of the changes: The second paragraph of the Introduction section.

Comment 2: Key genes (BECN2, VMP1, RUBCN) in Figure 1c should be highlighted to help readers focus on the critical points under discussion.

Author's reply: We thank the reviewer for this excellent suggestion. In response, we have now explicitly labeled the three key genes (BECN2, VMP1, RUBCN) in the legend of Figure 1c to help readers more easily identify and focus on these critical elements.

Location of the changes: The legend for Figure 1c (Page 18, Lines 368-369).

Comment 3: The representative IHC images in Figure 3c are predominantly stromal and lack clear mammary structures, such as mammary ducts. They should include images of more cellular regions to better demonstrate the cancer cell-specific expression.

Author's reply: We have replaced the representative IHC images in Figure 3c. The new images display clearer mammary duct structures and cancer cell-enriched areas, thereby more effectively illustrating the differential expression of RUBCN between cancerous and normal tissues.

Location of the changes: Fig 3.

Comment 4: The conclusion that RUBCN accelerates tumor progression by suppressing anti-tumor immunity may be somewhat premature and needs to be stated more cautiously. Furthermore, the specific breast cancer subtypes included in the analysis should be clearly specified.

Author's reply: We thank the reviewer for raising this important point. We agree that correlation does not directly imply causation. Accordingly, we have modified the relevant statements in the Discussion section (Page 28, Lines 577-586), changing the wording from a causal claim to a description of the observed correlation. In addition, we have now noted as a limitation of our study that the findings warrant validation across various molecular subtypes in the future.

Location of the changes: In the Discussion section.

Reviewer #2:

Comment 1: The second paragraph of the Introduction could be more concise, and the research objectives should be stated more explicitly.

Author's reply: We have streamlined and rewritten the introduction by removing redundant information, thereby more clearly articulating the study's objectives and the knowledge gap it aims to address.

Location of the changes: In the Introduction section.

Comment 1: How could RUBCN be used in diagnosis? Consider removing this section and conserving the content for the discussion on therapeutic applications.

Author's reply: We sincerely thank the reviewer for this astute observation. We completely agree that the discussion regarding the diagnostic potential of RUBCN was premature in the absence of specific diagnostic data. Following the reviewer's suggestion, we have removed the entire section speculating on diagnostic applications from the manuscript. The discussion is now focused and strengthened, emphasizing the therapeutic implications of our findings. We appreciate the reviewer's guidance, which has helped us present a more precise and rigorous interpretation of our results.

Location of the changes: Page 6, Lines 103-106.

Comment 2: It is recommended to perform a subgroup analysis of the TCGA data focusing on TNBC, and to provide clinical data support for the functional experiments conducted in TNBC cell lines.

Author's reply: We thank the reviewer for this valuable suggestion. Due to the limited sample size of TNBC cases in the TCGA cohort, we have acknowledged this as a limitation of our study in the Discussion section (Page 28, Lines 582-586), emphasizing the need for future validation in larger TNBC cohorts. Furthermore, we have added a justification for selecting TNBC cell lines (specifically MDA-MB-231) for our functional experiments, noting their higher expression levels, more aggressive phenotype, and limited treatment options, which collectively underscore the clinical relevance of investigating novel targets in this subtype.

Location of the changes: In the Discussion section.

Comment 3: It is strongly recommended to include additional Western blot analyses (e.g., for LC3-II and p62) to validate the changes in autophagic flux, thereby strengthening the mechanistic interpretation.

Author's reply: We sincerely thank the reviewer for this critical suggestion. Following the recommendation, we have performed the additional experiments. Upon RUBCN knockdown, we assessed the protein levels of LC3-II and p62 by Western blotting. The results revealed an increase in both LC3-II and p62 levels, indicating enhanced autophagic flux upon RUBCN knockdown. These new data provide crucial mechanistic support for our phenotypic observations regarding proliferation, migration, and invasion. The corresponding results have been included in the revised manuscript as Figure 9E and its accompanying description.

Location of the changes: A new paragraph describing the results and Figure 9E has been added to the Results section, and the details of the Western blot experiment have been updated in the Methods section.

Comment 4: The Methods section requires further clarification regarding specific experimental details, such as the number of spatial transcriptomics samples, the procedures for IHC and EdU assays, and the methodology used for image quantification.

Author's reply: We have thoroughly revised the Methods section to include all missing experimental details.

1. Specifically, the number of cases included in the spatial transcriptomics analysis has been clearly stated.
2. Detailed IHC experimental conditions as well as image acquisition and quantification methods have been added.
3. Detailed specifications for the EdU assay have been provided, including the incubation time, imaging methodology, and quantification process.
4. We agree with the reviewer and have corrected this error. The conflict of interest statement has been removed from the Methods section and is now appropriately placed as required by the journal's guidelines. Thank you for highlighting this.
5. We thank the reviewer for highlighting the need for this important methodological detail. As suggested, we have now explicitly stated in the main text of the Methods section that all Western blot experiments were performed in triplicate. The corresponding data from these independent replicates are included in the supplementary materials.
6. We thank the reviewer for the comment. Figure 9D was quantified by counting the number of invaded cells. The x-axis represents the experimental groups (siCtrl vs. siRUBCN) and therefore has no unit, which we have now clarified in the figure legend. The corresponding quantification method has been added to the Methods section.
7. We confirm that all catalog numbers have now been consistently listed in the Methods section.

Location of the changes: The relevant subsections within the Materials and Methods section.

Comment 5: The reviewer has provided specific suggestions for improving the presentation and labeling of several figures.

Author's reply: We fully appreciate the reviewer's intention to further demonstrate inter-sample heterogeneity through PCA. However, the objective of this specific part of our study was primarily to illustrate the feature importance (i.e., model coefficients) of candidate autophagy-related genes, as determined by multiple machine learning algorithms. Figure 3A, as a heatmap, is specifically designed to visualize the distribution and consistency of these coefficients across different algorithms during the feature selection process, rather than for sample clustering.

Therefore, we have retained the heatmap as the optimal format for presenting feature importance. Regarding the number of genes, 7 out of the initial 46 genes were automatically excluded by the algorithmic filtering process for the following reasons:

1. Their expression levels were extremely low (close to zero or constant) in the public training dataset;

2. They contained missing values in a subset of samples, making them unsuitable for feature calculation;

3. They were zeroed-out by the model's regularization process.

Consequently, the final model incorporated 39 genes, whose coefficients are visualized. This filtering is an inherent part of robust feature selection and does not compromise the overall stability or predictive performance of the model.

The IHC images in Figure 3c have been updated to include clearer epithelial regions and ductal structures. A scale bar has been added to the figure legend, and the image contrast has been optimized to better distinguish specific staining from background. These changes have been implemented in the revised manuscript.

We have clarified the criteria for classifying the high- and low-elasticity network groups in the Materials and Methods section (Page 9, Lines 163-165).

Comment 6: Specific suggestions were provided to improve the presentation and labeling of multiple figures (e.g., Figure 6), including ensuring formatting consistency, adding clear labels, refining panel 6E, and strengthening the logical integration between the figures and the text.

Author's reply: We sincerely thank the reviewer for these meticulous and valuable suggestions. We have thoroughly revised Figure 6 according to the recommendations provided.

We thank the reviewer for these important technical comments. We have now added scale bars to all relevant images. The specific image in panel 8E has been re-acquired to ensure clarity, and the label "Data 3" in panel 8F has been removed as suggested. These corrections have been implemented in the revised figure.

We sincerely thank the reviewer for their insightful and constructive comments, which have significantly strengthened the discussion of our findings. We have carefully addressed each point as follows:Regarding the causal interpretation of the RUBCN-immune interface, we have revised the relevant statements in the Discussion section (Page 28, Lines 577-586). The wording has been changed from a causal assertion to a description of the observed correlations, replacing the claim that "RUBCN accelerates tumor progression by suppressing antitumor immunity" with a more precise and nuanced description of the observed negative correlations. We have explicitly stated that the exact mechanistic relationship requires future functional validation of the immune landscape.Concerning clinical implications and limitations, we have expanded the discussion to include the study's limitations and to explore more deeply the potential clinical significance of RUBCN, including its possible role as a predictive biomarker or therapeutic target.We believe these revisions substantially enhance the balance, clarity, and translational relevance of the Discussion section.
