# Supplementary material for: RUBCN as a novel prognostic biomarker and therapeutic target in breast cancer
Source: PLoS One. 2026 Jan 27;21(1):e0341357. doi: 10.1371/journal.pone.0341357 (PMC12843558; doi:10.1371/journal.pone.0341357)
Supplement: Attachment — Submitted filename: Response_to_Reviewers_auresp_2.docx [file pone.0341357.s018.docx]

Title of the manuscript: RUBCN as a Novel Prognostic Biomarker and Therapeutic Target in Breast Cancer

Manuscript Number: PONE-D-25-46002

Dear Dr. Amy McCart Reed and Reviewers,

We are pleased to submit the revised version of our manuscript titled “RUBCN as a Novel Prognostic Biomarker and Therapeutic Target in Breast Cancer”. We sincerely thank you for handling our manuscript and for the constructive comments provided by the reviewers. We have carefully addressed all points raised in the previous round of review. The revisions have been made accordingly in the manuscript, and our point-by-point responses are detailed below. We believe the manuscript has been significantly improved and hope it now meets the journal’s standards for publication. Thank you for your time and consideration.

Reviewer #1:

We thank the reviewer for the valuable feedback. All points raised have been addressed below, and we believe these changes have strengthened the manuscript.

Comment #1: One last thing that needs to be added is in figure 9E, where the western blot is done on protein extracted from RUBCN KD MDAMB231s but I cannot see RUBCN expression as part of that blot- I think it’s important to have an additional blot from this experiment for RUBCN expression for a complete story.

Author's reply: We sincerely thank the reviewer for the positive feedback and for raising this important point. To provide a complete and rigorous validation of the knockdown model in this experiment, we have now performed the additional Western blot to assess RUBCN expression using the same protein samples from RUBCN-knockdown MDA-MB-231 cells. As suggested, the new RUBCN blot has been added to Figure 9E. This addition strengthens the figure by directly confirming the knockdown efficiency in the precise samples used for assessing autophagic flux, thereby providing a more complete story as suggested.

We are grateful for this insightful suggestion, which has undoubtedly improved the clarity and validity of our data.

Reviewer #2:

We thank the reviewer for the positive assessment of our revisions and for these additional constructive suggestions, which have helped us further improve the clarity and robustness of the manuscript. We have addressed all points as detailed below.

Comment #1: At line 68, please add “basal-like” after TNBC for clarity. Throughout the manuscript, please also remove all “—” symbols, as these appear to be auto-generated characters rather than standard punctuation.

Author's reply: We thank the reviewer for the suggestion. We have added "basal-like" after "TNBC" on line 68 for better clarity. Additionally, as recommended, we have removed all instances of the long dash ("—") throughout the manuscript and replaced them with standard punctuation.

Key locations of these changes include: Page 3, Lines 36-38; Page 5, Lines 70-73; Page 20, Lines 417-418.

Comment #2: At line 244, the Methods state that non-specific binding was blocked with 5% bovine serum albumin for 30 minutes; please clarify what the 5% BSA was diluted in. At line 301, please specify the manufacturer of Abmart.

Author's reply: We apologize for the omission. The 5% BSA was diluted in phosphate-buffered saline (PBS). We have clarified this in the revised Methods section.

Location of the changes: Page 13, Lines 246-248.

Comment #3: At line 301, please specify the manufacturer of Abmart.

Author's reply: We have now specified the manufacturer for the anti-LC3 antibody from Abmart in the revised text.

Location of the changes: Page 15, Lines 305.

Comment #4: For Figure 3C, it would be helpful to include representative images demonstrating staining intensity scores (0–3+). Please also clarify whether the graph represents a combined score and, if possible, provide a breakdown of staining intensity across normal and tumour tissues. In addition, please confirm that all images are at 100× magnification, as there appear to be differences in cell size across panels.

Author's reply: We sincerely thank the reviewer for the insightful suggestions, which have helped us improve the clarity of our manuscript. We have addressed each point as follows.

1. Clarification of the scoring method and confirmation of magnification:

As detailed in the Methods section, we used a semi-quantitative scoring system evaluating both intensity (0-3) and extent (0-5), with a final combined score ranging from 0 to 8. To prevent any ambiguity, we have now explicitly stated in the revised legend of Figure 3C: "Data are presented as the combined immunohistochemistry score (staining intensity score + extent score)." We confirm that all representative photomicrographs in Figure 3C were acquired at a consistent 100× original magnification.

Location of the changes: Page 20, Lines 410-413.

2. Regarding the request for a breakdown of staining intensity:

We appreciate the reviewer's suggestion to provide a more granular view of the intensity distribution. Our semi-quantitative analysis was designed to yield the validated combined score (0-8) as the primary metric for statistical comparison, as described. The scoring was performed directly to assign this final composite value per sample. We acknowledge that presenting the separate distribution of the four intensity sub-categories (0, 1+, 2+, 3+) would offer an additional perspective. However, to ensure scoring consistency and minimize intra-/inter-observer variability, the original data were recorded and analyzed as the integrated final score. A retrospective, accurate re-analysis to disaggregate the contribution of each intensity level across all samples is not feasible without introducing potential bias and is beyond the scope of the current revision.

3. Validation of the presented data:

We assure the reviewers that the data provided strongly supports our conclusion. The comparison of staining intensities between the "Normal" and "Tumor" panels in Figure 3C can visually confirm the significant increase in the comprehensive score of the tumor tissue. Tumor images uniformly show stronger and more extensive staining, corresponding to higher intensity (2+ or 3+) and range scores.

We believe that the current method and presentation employ a standard and transparent scoring system, providing clear and conclusive evidence for differential protein expression. Thank you to the reviewers for your valuable suggestions. We will take these suggestions into account in the design of future research.

Comment #5: In the Results section, when presenting immunohistochemistry data, please use caution when describing “upregulated expression” and clearly explain how the quantitative IHC results were derived.

Author's reply: We agree with the reviewer and have revised the description to be more precise. The revised text now avoids the term “upregulated” and explicitly states that the comparison is based on quantitative H-score analysis of paired samples. The updated text in the Results section reads: “To evaluate RUBCN expression at the protein level, we performed immunohistochemical (IHC) analysis on five paired samples of primary breast cancer and adjacent normal tissues. Quantitative IHC scoring revealed that RUBCN protein levels were significantly higher in tumor tissues compared with their paired normal counterparts (Figure 3C).”

This revision provides a clearer and more accurate description of the IHC data and its quantification.

Location of the changes: Page 20, Lines 401-405.

Comment #6: Please include the RUBCN antibody validation using the knockout control in Figure 9E to further support the specificity and robustness of these findings.

Author's reply: This is an excellent suggestion. We have now performed the recommended control experiment. Specifically, we conducted an additional Western blot using the same protein lysates from RUBCN-knockdown MDA-MB-231 cells to verify the knockdown efficiency. The updated Figure 9E now includes this RUBCN blot, which confirms the successful knockdown. The incorporation of this validation data strengthens the specificity of our experimental approach and the robustness of the corresponding conclusions. We sincerely appreciate the reviewer's insightful comment.
